# Convergence of carbapenem resistance and hypervirulence in a highly-transmissible ST11 clone of *K. pneumoniae*: An epidemiological, genomic and functional study

**DOI:** 10.1080/21505594.2020.1867468

**Published:** 2021-01-18

**Authors:** Ping Li, Qiqiang Liang, Wugao Liu, Beiwen Zheng, Lizhang Liu, Wei Wang, Zhijiang Xu, Man Huang, Youjun Feng

**Affiliations:** a Department of Pathogen Biology & Microbiology and Department of General Intensive Care Unit of the Second Affiliated Hospital, Zhejiang University School of Medicine, Hangzhou, Zhejiang China; b Clinical Laboratory of Lishui People’s Hospital, Lishui, China; c The First Affiliated Hospital, Zhejiang University School of Medicine, Hangzhou, China; d Clinical Laboratory, the Second Affiliated Hospital, Zhejiang University School of Medicine, Hangzhou, China; e Non-coding RNA and Drug Discovery Key Laboratory of Sichuan Province, Chengdu Medical College, Chengdu, China; f College of Animal Sciences, Zhejiang University, Hangzhou, China

**Keywords:** Whole-genome sequencing, Infection genomics, hypervirulence, Carbapenem resistance, *Klebsiella pneumoniae*, ST11, Colonization

## Abstract

Co-occurrence of hypervirulence and KPC-2 carbapenem resistant phenotypes in a highly-transmissible ST11 clone of*Klebsiella pneumoniae* has elicited deep concerns from public health stand point. To address this puzzle, we conducted a large-scale epidemiological, clinical and genomic study of *K. pneumonia* ST11 clones with both hypervirulence and carbapenem resistance in two tertiary hospitals in Zhejiang province. Most of the patients (15/23) were diagnosed with exclusively carbapenem-resistant *K*. *pneumoniae *(CRKP) infections. Ten death cases were reported, some of which are due to the failure of antibiotic therapies. As a result, we identified one new rare sequence types (ST449) to KPC-2-producing CRKP, in addition to the dominant ST11. These clinical isolates of *K. pneumoniae* are multi-drug resistant and possess a number of virulence factors. Experimental infections of wax moth larvae revealed the presence of hypervirulence at varied level, suggesting the complexity in bacterial virulence factors. However, plasmid curing assays further suggested that the *rmpA2*-virulence plasmid is associated with, but not sufficient for neither phenotypic hypermucoviscosity nor virulence of *K*. *pneumoniae*. Intriguingly, all the *rmpA2* genes were found to be inactive due to genetic deletion. In total, we reported 21 complete plasmid sequences comprising 13 *rmpA2*-positive virulence plasmids and 8 *bla*_KPC-2_-harboring resistance plasmids. In addition to the prevalent pLVKP-like virulence plasmid variants (~178kb), we found an unexpected diversity among KPC-2-producing plasmids whose dominant form is IncFII-IncR type (~120kb), rather than the previously anticipated version of ~170kb. These findings provide an updated snapshot of convergence of hypervirulence and carbapenem resistance in ST11 *K. pneumoniae.*

## Introduction

*Klebsiella pneumoniae* is a Gram-negative facultative anaerobic bacterium that can be traced back to as early as 1882[1]. Clinically, it causes severe and deadly community-acquired and hospital-associated bacterial infections [2–4]. *Klebsiella pneumoniae* have evolved into two large sub-populations, namely the hypervirulent *K. pneumoniae* (hvKP) [5] and the carbapenem-resistant *K. pneumoniae* (CRKP) [4,6]. In general, hvKP is mainly restricted to the ST23 clonal background [7,8], which frequently results in life-threatening infections like pyogenic liver abscesses [8,9]. The presence of virulence plasmids [e.g.: pLVPK [10]] further suggests a phenotype of bacterial hypervirulence [11–13]. Most likely, these plasmids feature a set of genes encoding putative virulence factors. Namely, they include an aerobactin synthesis operon (*iucABCD*), the outer membrane ferric aerobactin receptor (*iutA*), a gene cluster of salmochelin production (*iroBCDN*), and regulator of mucoid phenotype A (*rmpA* and/or *rmpA2*) [11,13]. In contrast, the second group, CRKP, primarily belongs to a dominant ST11 clonal group in China [14–17], which causes untreatable nosocomial infections with a mortality risk of around 50% [18–20]. *Klebsiella pneumoniae* Carbapemase (KPC)-producing plasmids are the main genetic determinants for this phenotypic antimicrobial resistance [4,6,21–25]. Given its rapid spread, the KPC-2-harboring ST11 clone poses a substantial threat to clinical anti-infection therapy [15,20,25]. Not surprisingly, CRKP was listed by the World Health Organization (WHO) as a Priority I (CRITICAL) Pathogen in 2017 (https://www.who.int/en/news-room/detail/27-02-2017-who-publishes-list-of-bacteria-for-which-new-antibiotics-are-urgently-needed). In fact, a bigger and more worrisome concern now lies in the emergence and global dissemination of a possible superbug, an epidemic clone of *K. pneumoniae* in which hypervirulence and carbapenem resistance converge via horizontal transfer and evolution of plasmids[1].

Very recently, Chen and coworkers described five cases of lethal infections caused by KPC-2-bearing ST11 *K. pneumoniae* with hypervirulence in the Second Affiliated Hospital, Zhejiang University [26]. This suggested the emergence of a carbapenem-resistant hypervirulent clone of *K. pneumoniae* [27–29]. This finding was unexpected, but not without any precedent. Retrospectively, similar cases were reported in sporadic cases and referenced herein [22,30,31]. The genetic mechanism by which classic ST11 CRKP strains gain hypervirulence relies on the horizontal acquisition of a pLVPK-like virulence plasmid [1,16,26]. In this study, we attempted to bridge this knowledge gap. We have conducted a retrospective study of patients (23 in total) admitted to two different tertiary hospitals (namely the Second Affiliated Hospital and Lishui People’s Hospital) in the Zhejiang province of China, from 2016 to 2017. Combined string tests and experimental infections, our data of virulence plasmid curing suggests that *rmpA2* plasmid is associated with, but not sufficient for hypervirulence of *K. pneumoniae*, underscoring the complexity in virulence determinants rather than previously-recognized *rmpA2* plasmid alone. Along with the observation of Gu *et al* [26]., this study provides additional insights into the diversified convergence of hypervirulence and carbapenem resistance in the highly-transmissible ST11 clone of *K. pneumoniae*.

## Results

### Clinical description of CRKP-positive inpatients

**Figure 1.**
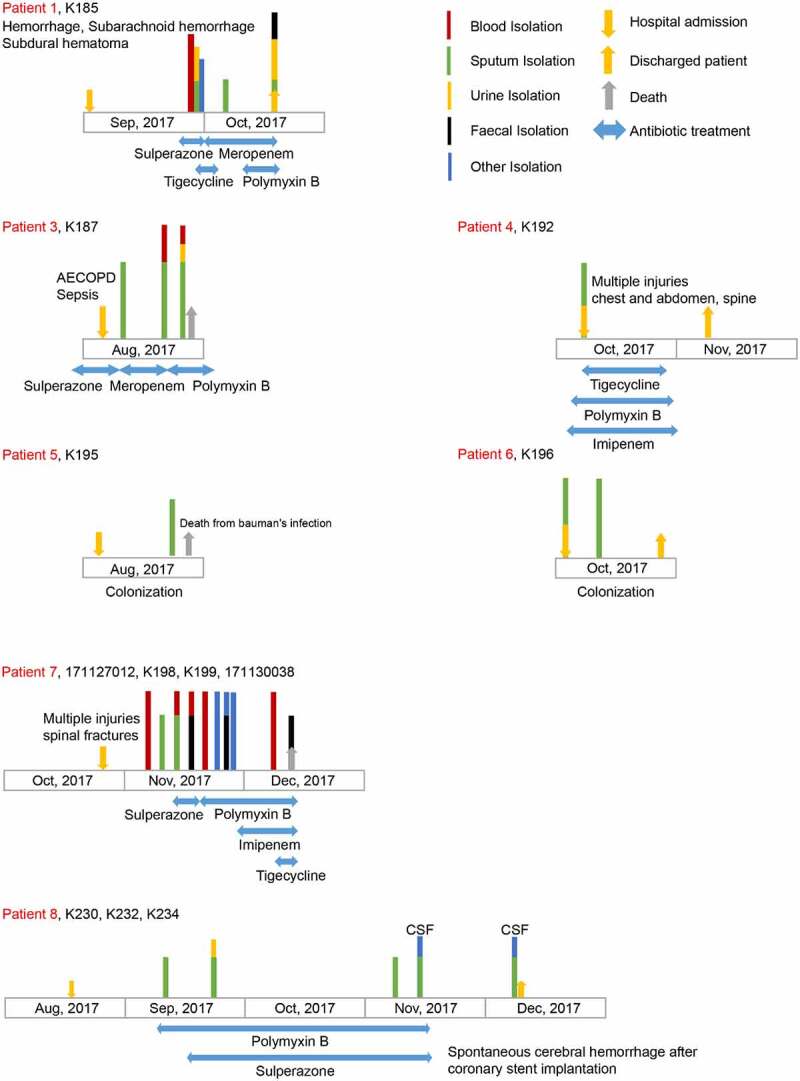
Description of clinical cases infected with *K. pneumoniae.*

Two tertiary hospitals in Zhejiang Province were involved in this clinical study, namely the Second Affiliated Hospital of Zhejiang University (Hangzhou City) and Lishui People’s Hospital (Lishui City, approximate 270 km south of Hangzhou City). In total, 23 patients (19 males & 4 females) that were admitted to the hospitals between late Dec. 2016 and Dec. 2017, were selected for this study (**Figure 1** and **S1-S3**). Among them, 17 patients were aged 16–80 years (15 males & 2 females) and admitted to the general ICU with single ward isolation (40 beds in-service of ICU with single ward isolation which treats approximately 1,500 patients each year) of the Second Affiliated Hospital of Zhejiang University, in Hangzhou City, from August to December of 2017 (**Figs S1-S2**). The remaining 6 patients (4 males & 2 females) were admitted in Lishui People’s Hospital, Lishui City, between late Dec. 2016 and Oct. 2017 (**Fig. S3**). These patients had suffered various forms of trauma caused by traffic accidents, falling objects, or acute spontaneous cerebral hemorrhages. Following varied surgeries, all the patients were subjected to antimicrobial treatment [either carbapenem alone or in the combination with other alternative antibiotics or mechanical ventilation (**Figure 1**, **S1-S3**) when necessary.

In the Second Affiliated Hospital of Zhejiang University, the length of hospital stays (LOS) of patients in ICU with single ward isolation varied from 0 days to 100 days during which six of 18 inpatients died. Among them, 7 individuals carried CRKP cultures in two or more sites, and the remaining 10 individuals were diagnosed with CRKP infections only in one site, five of which died (Supplementary Text and **Figs S1-S2**). As for the inpatients in Lishui People’s Hospital, the length of hospital stays varied from 5 days to one year. As expected, most of the patients developed pneumonia and CRKP strains were consistently isolated from each patient specimen (**Table S1**). Four out of six hospitalized patients suffered the failure of antibiotic treatment and died (**Fig. S3**).

In terms of treatment, the most common antibiotic regimens were combination therapies based on carbapenems. If the MIC value of carbapenems exceeded 16 in patients, carbapenems would not be considered. Tigecycline (in 9 cases) and polymyxin B (in 6 patients) were selected according to the patient’s situation and economic condition due to their high price while other options include amikacin, sulperazone, and fosfomycin. Patient 7’s condition rapidly deteriorated. After a bloodstream infection, the bacteria rapidly invaded the liver and gall bladder to form a liver and pericholecystic abscess. The patient developed DIC (Disseminated Intravascular Coagulation) and died rapidly. Finally, we successfully collected 19 CRKP isolates from 23 patients mentioned above (**Table S1**), including 5 positive sputum specimens, 5 positive urine samples, 3 positive blood samples, 2 positive feces samples and 4 other samples.

### Genetic characterization of CRKP

**Figure 2.**
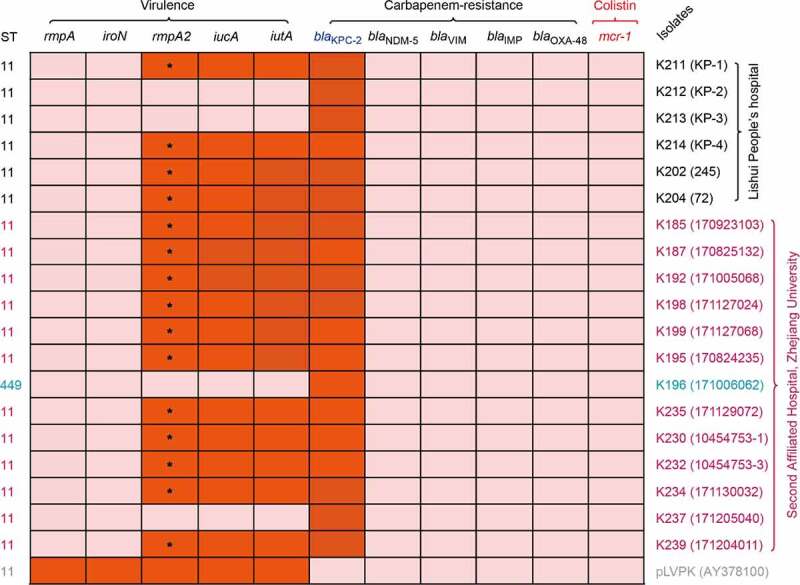
Investigation of the genes confering virulence and antibiotic resistance in *K. pneumoniae* Multiple PCR assays (**Table S5**) were applied to detect genetic determinants of virulence and antibiotic resistance in series of clinical strains (**Table S1**). The positive results of PCR are colored orange. * indicates the truncated version of *rmpA2*, which is due to the deletion – causing frameshift mutations and premature stop codons

Using MacConkey agar medium supplemented with imipenem, 19 non-redundant carbapenem-resistant *K. pneumoniae* strains of interest were recovered from the above clinical specimens (**Figure 1** and **Figs S1-S3**). Among them, 22 isolates of CRKP were sampled from 17 patients admitted into general ICU with single ward isolation, the Second Affiliated Hospital of Zhejiang University (**Tables S1-S2** & **Figs S1-S2**). Except for two patients who had multiple bacterial isolates each, i.e., 4 strains for ‘Patient 7ʹ and 3 strains for ‘Patient 8ʹ (**Figure 1**), all other inpatients were labeled with a single CRKP isolate (**Table S1-S2** & **Figs S1-S2**). The six isolates (one per patient) of CRKP from Lishui People Hospital separately corresponded to K202, K204, K211, K212, K213, and K214 (**Table S1** and **Fig. S3**). The identities of all these strains as *K. pneumoniae* were confirmed by both MALDI-TOF mass spectrometry and 16S rDNA sequencing. Antibiotic susceptibility assays revealed that they are consistently resistant to 6 classes of (10 different kinds of) antibiotics like Imipenem and Amikacin (**Tables S3-S4**), indicating that they are multi-drug resistant *K. pneuminiae*. The fact that they are all positive for *bla*_KPC-2_ in a PCR screen, allowed us to believe the phenotypic resistance to carbapenem is due to the presence of *bla*_KPC-2_ (**Figure 2**, **S6A** and **S7A**).

Given the results of PCR screening and sequence typing, 19 representative strains (13 strains from the Second Affilated Hospital of Zhejaing University, and 6 strains from Lishui People’s Hospital, in **Tables S1-S3**) were selected for genetic mapping and further genomic sequencing. Except that one strain is of non-ST11 genetic background (i.e., ST449 for K196), multilocus sequence typing (MLST) revealed that all the other 18 strains are exclusively grouped into ST11 (**Figure 2**). In general agreement with a recent observation by Gu *et al*. [26], ST11 was the dominant clone containing *bla*_KPC-2_ in hospitalized patients in these two hospitals during 2016–2017.

### Unexpected complexity in CRKP with hypervirulence

Even though the string test is a phenotype indicative of hypermucoviscosity, it is helpful, but not sufficient to conclude the correlation between phenotypic hypervirulence and hvKP strains [32]. Prior to string tests (**Figs S4A-F**), 6 representative hvKP isolates with *rmpA2*-positive plasmids were subjected to plasmid curing, which are confirmed with the *rmpA2*-, and KPC-2-specific PCR detection (**Fig. S4G**). These included four isolates (K185, K195, K199, and K235) from the Second Afiiliated Hospital, Zhejiang University, and two isolates (K204 and K214) are from Lishui People’s Hospital (**Tables S1** and **S4**). Among them, the only two strains are positive in string tests, namely K199 (50 mm, **Fig. S4C**), and K204 (40 mm, **Fig. S4D**). As predicted, plamid curing effectively impairs the phenotypic mucoviscosity of K199 and K204 (**Figs S4C-D** and **G**). Regardless of plasmid curing (**Fig. S4G**), all the other four hvKP strains remain negative in string tests, including K185 (**Fig. S4A**), K195 (**Fig. S4B**), K214 (**Fig. S4E**), and K235 (**Fig. S4F**), respectively.

**Figure 3.**
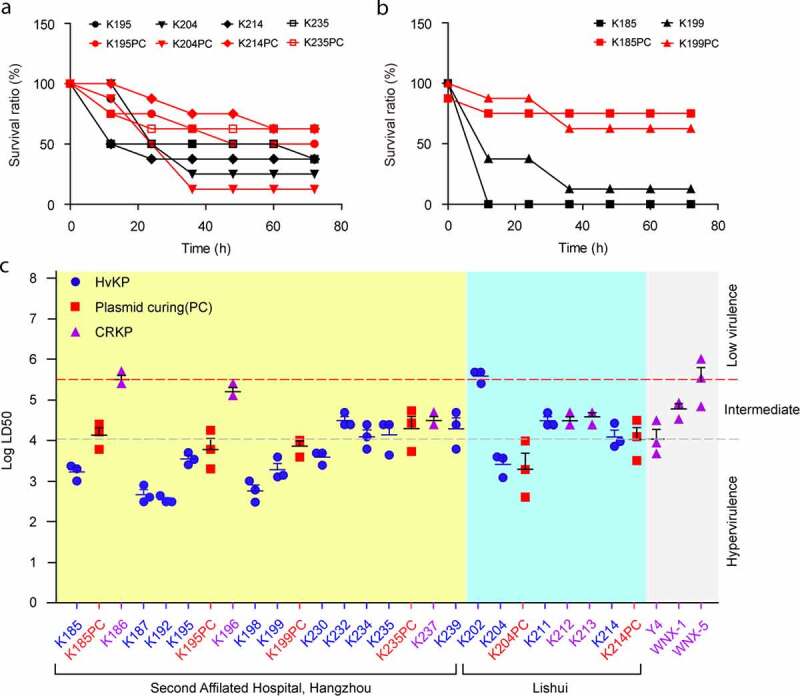
The use of *G. mellonella* infection model to evaluate the differentiation in virulence of clinical isolates of *K. pneumoniae* a. Plasmid curing has no significant effect on bacterial virulence of certain *K. pneumoniae* isolates (K195, K204, K214, and K235) b. The removal of virulence plamids from the two clinical isolates of *K. pneumoniae* (K185 and K199) attenuates bacterial pathogenicity To generate survival curves, *G. mellonella* is infected with 1 × 10^4^ cfu of individual *K. pneumoniae* isolate (black) and its plasmid curing derivative (red). c. Measurement of LD50 of clinical strains of *K. pneumoniae* in *G. mellonella* infection model The strains from Second Affiliated Hospital are indicated in yellow background, and strains from Lishui City are highlighted in cyan background. Three control strains are in gray background. Namely, they refer to Y4 strain with high virulence, WNX-1 with an intermediate virulence, and WNX-5 with the relatively-low virulene. The two baselines is indicated (i.e., red line is cutoff for low virulence and gray line is curoff for high virulence). PC denotes plasmid curing. CRKP (purple triangle), hvKP (black circle) and plasmid curing mutants (red square) have been evaluated. The data here suggests the strains/species-dependent role of virulence plasmids in various clinical *K. pneumoniae* isolates

Using a *G. mellonella* infection model (**Figure 3**), we observed that i) among the aforementioned six hvKP isolates (**Fig. S4**), only two strains (K185 and K199) can be significantly attenuated via the elimination of *rmpA2*-virulence plasmid by plasmid curing (Figure 3b), whereas not for the other four isolates (Figure 3a); and ii) LD50 of these *K. pneumoniae* were determined to vary from 10^2^ to 10^5^ (Figure 3c), suggesting various levels of virulence. Compared with WNX-5 (*rmpA/rmpA2* and aerobactin-negative), the negative control strain of low virulence, and CRKP, two viscous string-producing isolates (K199 and K204) and all the other string test-negative isolates (K185, K187, K192, K195, K198, and K230) exhibit a relatively-higher virulence (Figure 3c). Despite the carriage of *rmpA2*-virulence plasmid (**Figure 2**), certain clinical isolates (K211, K214, K232, K234, K235, and K239) exhibited intermediate virulence similar to those of the positive control isolate Y4 and virulence plasmids-negative CRKP isolates (K212, K213, and K237) (Figure 3c). In contrast, the eight hvKP isolates (K185, K187, K192, K195, K198, K199, K204, and K230) gave appreciable level of virulence (Figure 3c). Meanwhile, the hvKP strain of K202 is unusual in that its low virulence is relatively lower than most of the CRKP isolates lacking the virulence plasmids (Figure 3c). It is clear that no frank correlation occurs between *rmpA2* positivity and LD50. Surprisingly, although having lost the string test phenotype, strain K204-PC retains virulence in *Galleria* model. Therefore, this points out the complexity in phenotypic hypervirulence of hvKP, and suggests that *rmpA2* is not a single virulence determinant. Indeed, plasmid-borne aerobactin is also an important virulence factor.

### *Genomics of* rmp2*-harboring virulence plasmids*

The criteria applied in the selection of virulence plasmids candidate mainly relies on the distinct pattern of virulence determinants displayed in multiplex PCR assay (**Figure 2**). Therefore, 10 unique clinical *rmpA2*-positive isolates (**Fig. S5A**) were selected for further S1-PFGE analyses (**Fig. S5B**), together with Southern blot with the *rmpA2* probe (**Fig. S5C**). Among them, 6 isolates (K185, K187, K192, K195, K198, and K199) were collected from the Second Affiliated Hospital of Zhejiang University, and the other 4 strains (K202, K204, K211, and K214) are derived from the Lishui People’s Hospital (**Figure 2**). Similar to those reported by Gu *et al*. [26], our Southern blot analysis suggested an estimated size of 170–210 kb for the *rmpA2*-harboring plasmids (**Fig. S5C**). As expected, Illumina next-generation sequencing revealed that all the plasmid backbones (e.g., pK185-*rmpA2*) were around 178kb in length (**Table 1** and **S6**). Using Plasmid Finder 1.3 (https://cge.cbs.dtu.dk/services/PlasmidFinder-1.3/), we further confirmed that these *rmpA2*-containing plasmids belong to the “IncHI1B-IncFIB” replicon incompatibility type, analogous to those of the paradigm virulence plasmids pLVPK and pVir-CR-HvKP4 (**Table 1**).

**Table 1.** An overview of the sequenced plasmids/contigs from carbapenem-resistant, hypervirulent *K. pneumoniae* reported in this study

No.	Plasmids	Status	Types	Size (kb)	GC%	ORFs	Acc. no.
1#	pK185_KPC	Contigs	IncFII-IncR	~120	/	/	NA
	pK185_*rmpA2*	Complete	IncHI1B-IncFIB	178.268	50.46	215	MK347226
2#	pK187_KPC	Complete	IncFII-IncR	128.447	53.73	210	MK312241
	pK187_*rmpA2*	Complete	IncHI1B-IncFIB	178.537	50.49	215	MK347227
3#	pK192_KPC	Contigs	IncFII-IncR	~120	/	/	NA
	pK192_*rmpA2*	Contigs	IncHI1B-IncFIB	~180	/	/	NA
4#	pK195_KPC	Complete	IncFII-IncR	119.209	53.64	199	MK312242
	pK195_*rmpA2*	Complete	IncHI1B-IncFIB	178.038	50.41	233	MK347228
5#	pK196_KPC	Contigs	NA	~90	/	/	MK312243
6#	pK198_KPC	Contigs	IncFII-IncR	~120	/	/	NA
	pK198_*rmpA2*	Contigs	IncHI1B-IncFIB	~180	/	/	NA
7#	pK199_KPC	Complete	IncFII-IncR	118.895	53.49	191	MK312244
	pK199_*rmpA2*	Complete	IncHI1B-IncFIB	177.827	50.51	219	MK347229
8#	pK202_KPC	Contigs	IncFII-IncR	~120	/	/	NA
	pK202_*rmpA2*	Complete	IncHI1B-IncFIB	177.394	50.50	227	MK347230
9#	pK204_KPC	Complete	IncFII-IncR	119.860	53.81	198	MK312245
	pK204_*rmpA2*	Complete	IncHI1B-IncFIB	176.874	50.58	229	MK347231
10#	pK211_KPC	Southern blot	NA	~80	/	/	NA
	pK211_*rmpA2*	Complete	IncHI1B-IncFIB	178.605	50.54	231	MK347232
11#	pK212_KPC	Southern blot	NA	~60	/	/	NA
12#	pK213_KPC	Southern blot	NA	~60	/	/	NA
13#	pK214_KPC	Southern blot	NA	~180	/	/	NA
	pK214_*rmpA2*	Complete	IncHI1B-IncFIB	177.476	50.49	231	MK347233
14#	pK230_KPC	Complete	IncFII-IncR	120.581	54.17	196	MK312246
	pK230_*rmpA2*	Complete	IncHI1B-IncFIB	177.333	50.64	226	MK347234
15#	pK232_KPC	Complete	IncFII-IncR	118.504	53.68	197	MK312247
	pK232_*rmpA2*	Complete	IncHI1B-IncFIB	177.714	50.47	233	MK347235
16#	pK234_KPC	Southern blot	NA	~180	/	/	NA
	pK234_*rmpA2*	Complete	IncHI1B-IncFIB	178.421	50.50	233	MK347236
17#	pK235_KPC	Complete	IncFII-IncR	120.73	53.74	204	MK312248
	pK235_*rmpA2*	Complete	IncHI1B-IncFIB	177.43	50.59	230	MK347237
18#	pK237_KPC	Southern blot	NA	~60	/	/	NA
19#	pK239_KPC	Complete	IncFII-IncR	120.289	53.60	214	MK312249
	pK239_*rmpA2*	Complete	IncHI1B-IncFIB	178.156	50.54	243	MK347238

“NA”: not applied;/, not detected

A total of 19 clinical *K. pneumoniae* isolates were applied in plasmid isolations and the resultant plasmids of interest were subjected to Illumina next-generation sequencing. As a result, we assembled 21 complete plasmid sequences and 7 plasmid contigs (**Table 1** and **S6**). The completely assembled 13 *rmpA2*-positive plasmids and 8 KPC-2-producing plasmids (**Table 1**). Seven incomplete sequences corresponded to 2 large *rmpA2*-harboring fragments and 5 *bla*_KPC-2_-carrying contigs (**Table 1**). Not surprisingly, plasmid sequencing revealed that 15 clinical strains (such as K185 and K204) carry a *bla*_KPC-2_-positive plasmid along with a *rmpA2*-expressing plasmid (**Table 1**), which corroborated our PCR assays (**Figure 2**) and Southern blot results (**Figs S5-S7**). In addition, we demonstrated that 4 strains (namely K196, K212, K213, and K237) exclusively retain a single KPC-2-producing resistance plasmid, without the *rmpA2-*bearing virulence plasmid (**Table 1**), which is generally consistent with PCR-based virulence factor profiles (**Figure 2**).

**Figure 4.**
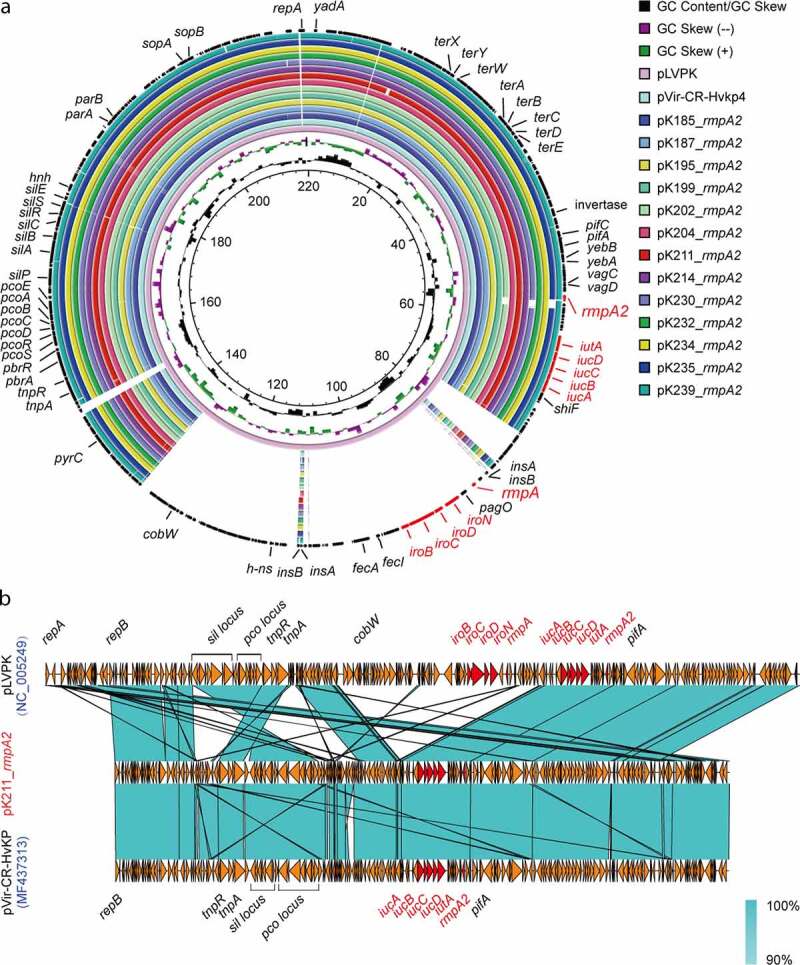
Comparative genomics of the *rmpA*-positive virulence plasmids from the ST11 carbapenemase-producing hypervirulent *K. pneumoniae.*

Comparative genomics of these *rmpA2*-positive plasmids showed that they consistently possess over 99% similarity (with 99% query coverage; Figure 4a) to pVir-CR-HvKP4 (Acc. no.: MF437313) and seem to be heterogeneous variants of this plasmid which was recently reported to be circulating in the Second Affiliated Hospital of Zhejiang University [26]. In addition to *rmpA2*, both *iucABCD* (an aerobactin synthesis operon) and *iutA* gene (that codes the outer membrane ferric aerobactin receptor) are also present in a number of sequenced plasmids (Figure 4a and b). Similar scenarios were also seen in other virulence plasmids detected in ST11 strains circulating in China [33,34]. This further validated the profile of virulence factors seen in our multiplex PCR assay (**Figure 2**). Even though they align well to the paradigm ~230kb long virulence plasmid pLVPK (Acc. no.: AY378100) [13], these virulence plasmids consistently lacked an approximately 50kb of region that carried the other mucoid regulator-encoding gene *rmpA* and *iroBCDN*, a gene cluster of salmochelin production (Figure 4b). These results provide genomic evidence for the discovery of epidemic virulence plasmids circulated in Hospitals of Zhejiang Province (**Figure 4**). Moreover, it seems likely that they are pLVPK-like derivatives generated by a genetic deletion event in response to an unknown selective pressure.

a. Genomic analyses of the thirteen pK211_*rmpA*-like virulence plasmids (roughly 178kb) recovered from ST11 carbapenem-resistant hypervirulent *K. pneumoniae*Genomic sequences of the above 13 plasmids were aligned against two known virulence plasmids (pLVPK of the *K. pneumoniae* strain CG43 [219kb, Acc. no.: AY378100] and pVir-CR-HvKP4 [178kb, Acc. no.: MF437313]).The circular map was generated with the BLAST Ring Image Generator. b. Colinear genome alignment amongst pK211_*rmpA2*, pLVPK (Acc. no.: AY378100) and pVir-CR-HvKP4 (Acc. no.: MF437313)The program Easyfig was applied in comparative genomics. Colored arrows indicate ORFs and the shaded region reflects sequence similarity. The virulence associated genes are indicated in red.

### *Diversity of* bla*_KPC-2_-bearing plasmids*

**Figure 5.**
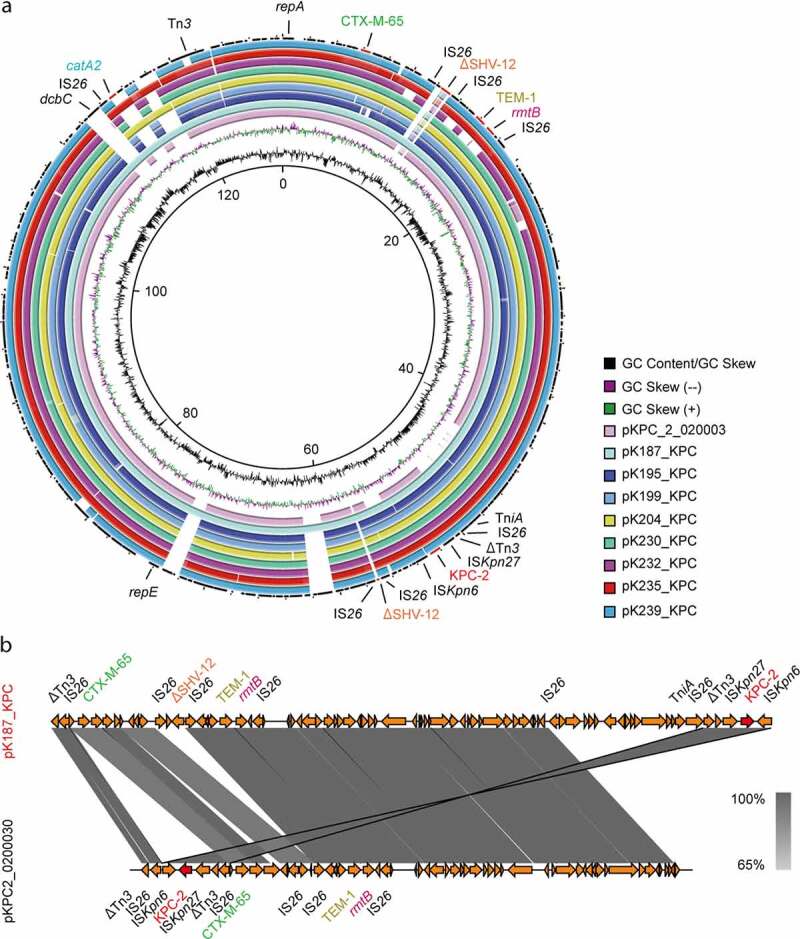
Comparative genomics of eight *bla*_KPC-2_-harboring plasmids isolated from clinical isolates of *K. pneumoniae.*

The *bla*_KPC-2_-specific molecular assays (PCR detection and Southern blot, **Figs S6-S7**) of 18 *K. pneumoniae* isolates showed clearly that at least 6 kinds of *bla*_KPC-2_-positive plasmids were disseminated in these clinical CRKP isolates, whose physical size ranges from ~10kb to ~130kb (**Table S6** and **Figs S6-S7**). Given that KPC-2 is transmitted by diversified plasmids in patients hospitalized in Zhejiang province, it is of much interest to gain genomic insights into these carbapenem resistance plasmids. As a result, plasmid sequencing returned 9 complete plasmid sequences (57.798–128.447kb) and 4 unclosed large contigs (120–180kb) of *bla*_KPC-2_-carrying plasmids (**Table 1**). Among them, the IncFII/IncR-type *bla*_KPC-2_-bearing plasmids (118.504–128.447kb) seemed to be prevalent and they were found to exist in strains that also carried the IncHI1B-IncFIB type, *rmpA2*-harboring hypervirulent plasmids (176.874–178.537kb) in 90% (8/9) of sequenced *K. pneumoniae* isolates (like K187, in **Table 1** and Figure 5a).

a. Genomic alignment of eight *bla*_KPC-2_-harboring plasmids (of rough 120kb) with pKPC_2_0200030 (Acc. no.: CP028790)The eight KPC-2-harboring plasmids reported in this study included pK187_KPC, pK195_KPC, pK199_KPC, pK204_KPC, pK230_KPC, pK232_KPC, pK235_KPC, and pK239_KPC, respectively. The circular map was generated with the BLAST Ring Image Generator. b. Colinear genome analyses of pK187_KPC and pKPC_2_0200030 (Acc. no.: CP028790)Easyfig was used for comparative genomic analysis. Colored arrows indicate ORFs and the shaded region depicts sequence similarity.

Sequence analysis and annotation indicated that the *bla*_KPC-2_-encoding plasmids carry additional antibiotic resistance determinants including extended-spectrum β-lactamase genes (*bla*_TEM-1_, *bla*_KPC-2_, *bla*_CTX-M-65_, *bla*_CTX-M-90_), aminoglycoside resistance gene (*rmtB*), phenicol resistance gene (*catA2*), etc. (**Figure 5** and **Table S7**). This finding can explain the phenotypic multidrug resistance of these clinical *K. pneumoniae* strains (**Tables S2-S3**). It is common that various mobile elements, mostly IS*26*, are located upstream and downstream of the aforementioned antimicrobial resistance genes (**Figure 5**) and might play a role in the AMR horizontal transfer. Genomic alignment of the eight IncFII/R type *bla*_KPC-2_-harboring plasmids suggested that they share nearly 99% identity with each other with query coverage ranging from 87% to 95% (Figure 5a). Although that they align well with a recently-recognized *bla*_KPC-2_-positive plasmid, pKPC2_0200030, from West China University (~105kb, Acc. no.: CP031720), our KPC-2-containing plasmids, like pK187_KPC, exhibit varied level of query coverage (85%-89%). This might hint that they are all longer versions with discontinuous inserts like the short plasmid pKPC2_0200030 (Figure 5a). Also, it is clear that a cassette surrounding *bla*_KPC-2_ (IS*Kpn26* – *bla*_KPC-2_-IS*Kpn27*-ΔTn*3*-IS*26*) is extremely conserved amongst these plasmids (**Figure 5**). However, linear genomic alignment pointed out that the insert direction in pK186_KPC and pKPC2_0200030 are opposite (Figure 5b). Presumably, it is due to active transposition between plasmids and highlights a risk that *bla*_KPC-2_ can spread across various isolates of ST11 *K. pneumoniae*.

### Discussion

The data shown here is in general agreement with the statement by Gu *et al*. [26] that i) occurrence of carbapenem resistance is conferred by KPC-2 in clinical isolates of *K. pneumoniae* in Zhejiang Province; and ii) the dominant KPC-2-producing *K. pneumoniae* belongs to ST11 in the Second Affiliated Hospital of Zhejiang University, 2016–2017. Despite our recent discovery of co-occurrence of the other carbapenemase-encoding gene *bla*_NDM-5_ the transferable colistin resistance determinant *mcr-1* on two distinct plasmids from the isolates of Lishui People’s Hospital [35], our multiplex-PCR screen cannot detect the co-occurrence of carbapenem-resistance gene *bla*_KPC-2_ with *mcr-1* in these clinical isolates. Of being noteworthy, this sampling in the same hospital, in 2017, allowed us to identify one rare sequence types (ST449) of *K. pneumoniae* carrying KPC-2 (**Figure 2**). More intriguingly, this unique isolates of *K. pneumoniae* (ST449 for strain K196, **Figure 2**) only carry a KPC-2-positive plasmid (~90kb for pK196-KPC in K196, **Fig. S6C**), but no virulence plasmids. It is plausible that the lack of virulence plasmid in the two strains (**Figure 2**), explains in part why the value of their LD50 is relatively-higher than certain hypervirulence plasmid-carrying strains like K187 and K192 (Figure 3c).

In general, an intact *rmpA2* gene is 639bp long, which encodes RmpA2 regulator of 212aa in full length. Unlike the scenario seen with pVir-CR-HvKP4 having the intact *rmpA2* version [26], Sanger sequencing elucidates that an identical-truncated version of *rmpA2* consistently occurs in all the 15 isolates collected in this study (**Fig. S8**). Along with an “A” deletion in the poly-tract at the position 353, the two “G” deletion in the poly-G tract at position 285–286 (**Fig. S8A&B**), results in the frameshift and premature stop codon, giving a predicted polypeptide product of 99 amino acids (**Fig. S8C**). Because of lacking DNA-binding domain at C-terminus, the truncated version of RmpA2 is presumably nonfunctional. Not surprisingly, the discovery of such *rmpA2* variant appears in our bacterial species. This is because that it has already been recorded in NCBI database, involving 40 other *K. pneumoniae* isolates. Among them, 37 isolates arise from China (e.g.: 13 isolates from Hunan Province, China [Acc. no.: PRJNA638288, MT090958, and MT035874] [36] and the isolate NTUH-K2044 from Taiwan [Acc. no.: AP006726] [37]). The remaining three isolates are separately derived from USA (Acc. no.: CP037743), Japan (Acc. no.: AP019549), and South Korea (Acc. no.: CP030924). The functional inactivation of *rmpA2* might indicate an ongoing microevolution of *K. pneumoniae* colonizing in Zhejiang Province, China.

Our experimental infections with wax moth larvae confirmed that bacterial virulence differ across these isolates (**Figure 3**). Several *rmpA2*-negative CRKP isolates (like K212, K213, and K237) gave comparable intermediate virulence in relative to certain HvKP strains (e.g., K211, K232, and K239) (Figure 3c). Of note, *Galleria* possibly is not the best model to test the relationship between hyper-mucoviscosity and virulence. We only found 10% (2/20) of the representative HvKP isolates are positive in string test, which is quite lower than 100% (5/5) in a recent report of Gu *et al*. [26]. This argues that *rmpA2*-virulence plasmid is a necessary, but not sufficient for the formation of hyper-mucoviscosity, a virulence-associated phenotype. Not surprisingly, the *rmpA2*-virulence plasmid curing diminished the phenotypic hyper-mucoviscosity in the string tests for the two strains K119 and K204 (**Figs S4C-D**). However, the elimination of *rmpA2*-virulence plasmid only attenuated virulence of K199 (Figure 3b-**C**), rather than K204 (Figure 3a and **C**). This underscored that *rmpA2* virulence plasmid can’t be a sole determinant of hypervirulence in *K. pneumoniae*. Moreover, the fact that bacterial hypervirulence varies greatly amongst different virulence plasmids-bearing isolates allowed us to believe that the role of virulence plasmid is strain-dependent and associated with the context of *K. pneumoniae* isolates. Because that i) hypervirulence is a complex phenotype associated with but not limited to hyper-mucoviscosity, (i.e., hyper-mucoviscosity is not synonmous with hypervirulence in certain *rmpA2*-bearing *K. pneumoniae* [38]); and ii) hypermucoviscosity is dependent on production of biofilm, siderophores (especially aerobactin), fimbriae adhesion, and polysaccharides, we further speculated that our isolation might represent a heterogeneous/diversified population of HvKP. In fact, this hypothesis was supported at the level of plasmids since most (if not all) were harbored in these clinical isolates (Figure 2–5). In general consistence with the scenario assigned to HvKP4 described by Gu and coworkers [26], we found that 75% (15/20) of our *K. pneumoniae* isolates coharbors both a *rmpA2*-virulence plasmid and a carbapenem-resistance plasmid (**Table 1** and **Figure 2**). Indeed, all the hypervirulence plasmids we determined here, are ~178 kb in length, and possess 99% identity to pVir-CR-HvKP4 (Acc. no.: MF437313) reported by Gu *et al*. [26]. This indicated that they are a series of shortened variants from the original virulence plasmid, pLVPK (~219 kb, **Figure 4**) [13]. Therefore, we concluded that the plasmid determinants of hypervirulence are relatively-conserved, but retain the potential for microevolution in current isolates of CRKP in Zhejiang’s hospitals.

In contrast, the KPC-2-harboring plasmids with carbapenem resistance displayed an unexpected diversity (**Table 1** and **Figure 5** & **S6-S7**). We also noted that the dominant form of *bla*_KPC-2_-carriers in Zhejiang’s hospitals is IncFII/IncR-type plasmid with the size of ~120kb (**Table 1**), rather than ~178kb IncFII/IncR-type reported by Gu *et al*. [26]. Given that a polyclonal ST11-KPC plasmid scenario is observed, we anticipated that several parallel events of *rmpA2* plasmid acquisition might have occurred, giving rise to at least 2 clones ST11-KPC-*rmpA2* clones, rather than the only one described by Gu *et al* [26]. In addition, 8 resistance plasmids with complete plasmid sequences are localized at the size ranging from 118.504kb to 128.447kb (**Table 1**). Obviously, the complexity of KPC-2-positive plasmids in ST11 CRKP supplemented the conclusion of Gu *et al*. [26] that the emergence of ST11 carbapenem-resistant hypervirulent *K. pneumoniae* is due to a single genetic event, an acquisition of a pLVPK-like virulence plasmid (~170kb) by a classic ST11 CRKP presumably carrying a dominant one pKPC-CR-HvKP4 (~170kb). Probably, it has already arisen purely through chance and continues to circulate due to improved fitness costs. It is unusual that *bla*_KPC-2_ coexists with *rmpA2* in a hybrid plasmid pKP70-2 (~240kb) from HvKP [39]. Evidently, it might be more complicated than we earlier anticipated.

### Conclusions

Very recently, the discovery that *rmpA2*-harboring plasmid hypervirulence converges with KPC-2 carbapenem resistance in a highly-transmissible ST11 clone of *Klebsiella pneumoniae* raised concerns among public health professionals and social communities [26]. However, the description of CR-HvKP isolations is incomplete. This is primarily due to the limited availability of clinical cases and genomic sequences of the causative agents. To address this issue, we conducted a large-scale epidemiological, clinical, and genomic study in two tertiary hospitals in Zhejiang Province. Overall, our finding provides additional insight into the diversity of CR-HvKP isolates in Zhejiang province. 7 new major discoveries are described as follows: i) 23 patients admitted to hospitals were clinically described, most of which had undergone surgery for various traumas and then subjected to antimicrobial treatment of carbapenem alone (or combined with other alternative antibiotics); ii) we discovered one new rare sequence types (namely ST449), in addition to the prevalent ST11 sequence type; iii) as for these newly-isolated KPC-2-producing CRKP, they all displayed the complexity of multidrug resistance as well as genetic heterogeneity in virulence factor profiles; iv) to gain detailed genomic insights into the convergence of hypervirulence and carbapenem resistance, we completed plasmid sequencing of 21 plasmids consisting of 13 *rmpA2*-positive virulence plasmids and 8 *bla*_KPC-2_-harboring resistance plasmids; v) the rmpA2 regulator were found to be inactive variants here; vi) we observed that all the virulence plasmids are pLVKP-like variants of ~178kb long, and proposed the complexity of plasmid-encoded virulence factors in the context of *K. pneumoniae* hypervirulence; and vii) the unexpected diversity among these KPC-2-producing resistance plasmids were identified, whose dominant form belongs to IncFII-IncR type plasmid of ~120kb, not the previously-anticipated version of ~170kb. In summary, our findings benefit our understanding on the convergence of hypervirulence and carbapenem resistance in ST11 *K. pneumoniae*, providing additional genomic insights into the diversity of dominant clones in Zhejiang province.

## Methods

### Study design

In 2017, a small-scale outbreak of carbapenem-resistant hypervirulent *Klebsiella pneumoniae* (CR-hvKP) was recorded in an integrated ICU of the Second Affiliated Hospital of Zhejiang University (Hangzhou, China). We therefore intended to investigate whether CR-hvKP infections were becoming increasingly prevalent among ICU patients. Overall, clinical strains included in this study referred to carbapenem-resistant *Klebsiella pneumoniae* (CRKP) strains sampled by clinical laboratories from patients admitted to two independent ICUs (the Second Affiliated Hospital of Zhejiang University, and Lishui People’s Hospital), from Dec. 2016 to Dec. 2017.

### Ethics statement

Clinical data were recorded and approved by the local ethics committee from both the Second Affiliated Hospital, Zhejiang University School of Medicine (2018–01-F) and the Lishui People’s Hospital (2017–005-01).

**Clinical Isolates, Identification and Growth Condition**

All the clinical isolates of *Klebsiella pneumoniae* (*K. pneumoniae*) were collected from the patients admitted to two public Grade-A Tertiary Hospitals in Zhejiang Provinces, China. This includes the Second Affiliated Hospital, Zhejiang University, and Lishui People’s Hospital, Lishui City, respectively. Prior to direct sequencing of 16S rDNA PCR products, bacterial identification was conducted routinely using matrix-assisted laser desorption/ionization-time of flight mass spectrometry (MALDI-TOF MS) [40]. The isolated *K. pneumoniae* (**Table S1**) were cultivated overnight on either MacConkey agar media [or Luria-Bertani Broth (LB) agar plates] or LB liquid medium at 37°C for further sequence typing and plasmid isolation.

### Assays for antimicrobial susceptibility

The *K. pneumoniae* strains of interest were subjected to antimicrobial susceptibility tests comprising 13 different antibiotics such as Imipenem and Ertapenem (**Tables S3-S4**). The minimum inhibitory concentration (MIC) was routinely determined in accordance with the general guidance of Clinical and Laboratory Standards Institute. The MIC values were interpreted in terms of the breakpoints recommended by the European Committee on Antimicrobial Susceptibility Testing (EUCAST) [41,42].

### String test

To address the variation of phenotypic muco-viscosity, an indication of bacterial virulence, all the *K. pneumoniae* isolates (**Table S1**) were inoculated on Columbia blood (5%) agar plates (Oxoid, Thermo Scientific) and maintained at 37°C overnight. In general, a toothpick was used to touch and pull a single colony upwards. The cutoff criteria of being positive in the string tests denotes the >5 mm length of a vicious string.

### Multi-locus sequence typing

Genetic diversity of *K. pneumoniae* was examined with multi-locus sequence typing (MLST). In general, 7 sets of DNA fragments derived from house-keeping genes were PCR amplified with specific primers (**Table S5**), as routinely described in the protocol-specific in the online MLST database for *K. pneumoniae*. The gel-purified PCR products were subjected to further DNA Sanger sequencing. Lastly, the sequence data with various allelic profile was submitted to the MLST database (https://www.pasteur.fr/fr/mlst), giving final sequence type.

### PCR-aided search for virulence and antibiotic resistance genes

Multiplex PCR systems were established to detect the putative genes associated with either virulence or carbapenem resistance in *K. pneumoniae*. The five loci of hypervirulent-*K. pneumoniae* (hvKp) we examined here referred to *rmpA* (regulator of mucoid phenotype A), *iroN, rmpA2* (an activator for capsule biosynthesis), *iucA*, and *iutA*, respectively (**Table S2**). In addition to the mobile colistin resistance gene *mcr-1* [43], five distinct genetic determinants encoding carbapenemases were also detected (**Table S2**), namely *bla*_KPC-2_, *bla*_NDM-5_, *bla*_VIM_, *bla*_IMP_, and *bla*_OXA-48_ [23]. After visualization of these PCR products separated with agarose (1%) gel electrophoresis, they were further confirmed by direct DNA sequencing.

### S1-PFGE and southern blot

To reveal physical location of the *bla*_KPC-2_ (and/or *rmpA2*) determinant in clinical isolates of *K. pneumoniae*, S1-Pulsed Field Gel Electrophoresis (S1-PFGE) was conducted, along with Southern blot as recently described [44,45]. Overnight culture of different *K. pneumoniae* strains (**Table S1**) was imbedded in 1.0% agarose gel plugs (at the ratio 1:1), which was followed by the protease K treatment. Subsequently, *S1* nuclease (Takara, Dalian, China) was applied to treat agarose gel plugs, prior to the separation of DNA fragments by PFGE [44,45]. Then, Southern blot was routinely performed in accordance with the manufacturer’s protocol. In particular, two types of probes used here referred to digoxigenin-labeled PCR products separately targeting *bla*_KPC-2_ and *rmpA2*.

### Plasmid curing

To determine potential effects of virulence plasmids on *K. pneumoniae* infection (**Table S6**), plasmid curing was conducted with the treatment of sodium dodecyl sulfate (SDS) as described by El-Mansi *et al*. [46]. Six *K. pneumoniae* isolates carrying *rmpA2*-positive virulence plasmids were examined here, which included four strains (K195, K204, K214, and K235) from Second Affiliated Hospital, Zhejiang University, and two isolates (K185 and K199) from Lishui People’s Hospital (**Table S1**). Prior to plasmid curing, various level of SDS (from 0.5%, 2.5%, to 5.0%) was supplemented into liquid LB culture and 2 μg/ml meropenem was added into LBA plates. In brief, 100 μl of log-phage culture was inoculated into 10 ml of liquid SDS-containing LB media, and kept overnight with shaking at 27°C. Then, the resultant culture was diluted, plated on the meropenem-selecting LB agar plates, and incubated overnight at 37°C. Finally, single colonies were subjected to genetic screening of the plasmid-curing derivatives with *rmpA2* – and *bla*_KPC-2_-specific PCR assays (**Table S5** and **Fig. S4**).

### Galleria mellonella *infection*

The virulence of *K. pneumoniae* was evaluated using the infection model of wax moth larvae (*Galleria mellonella*) as described earlier [47,48]. *G. mellonella* (~300 mg each) was purchased from Tianjin Huiyude Biotech Company (Tianjin, China), and divided into series of experimental groups (10/group). Larvae was challenged with log-phase cultures of *K. pneumoniae* at appropriate level of colony-forming units (CFU). As for survival curves, *G. mellonella* were infected with 10^4^ CFU of *K. pneumoniae* and recorded 72 h post-infection. To further measure LD50 (lethal dose 50%), larvae was injected with different concentrations of *K. pneumoniae* ranging from 10^3^, 10^4^, 10^5^, to 10^6^ CFU. All the experimental infections were conducted in triplicates.

### Plasmid sequencing and assembly

Plasmids were extracted from clinical *K. pneumoniae* isolates using the Qiagen plasmid Midi-prep kit (Qiagen, Germany). After quality control on agarose gel, plasmids were used to construct a DNA library using KAPA Hyper Prep Kit used for Illumina platform (Roche, Basel, Switzerland). The resultant libraries were sequenced with the Hiseq X ten PE150 sequencer platform (Illumina, USA), giving a pool of 150 bp paired-end reads. The reads were trimmed and then assembled into a contig by the SPAdes Genome Assembler (version 3.11.0). A BLASTN against every contig containing resistance (and/or virulence) gene was carried out to find out best-hit plasmids. In light of the best-hit plasmids, possible gaps between contigs were closed with PCR combined with Sanger sequencing. All the plasmids with complete plasmid sequences have been deposited into GenBank (**Table 1**).

### Sequence annotation and genome comparison

Open reading frames (ORFs) were predicted using RAST (rapid annotation using subsystem technology, http://rast.nmpdr.org) combined with BLASTP/BLASTN searches. The plasmid maps were generated using GenomeVx (http://wolfe.ucd.ie/GenomeVx/). The comparison of circular plasmid map was given with the BLAST Ring Image Generator (version 0.95) [49], whereas linear alignments of multiple genomic loci were conducted using Easyfig [50]. Plasmid incompatibility typing were identified by PlasmidFinder 1.3 (https://cge.cbs.dtu.dk/services/PlasmidFinder-1.3/). Antibiotic resistance genes were detected by ResFinder 3.1 (https://cge.cbs.dtu.dk/services/ResFinder/).

## Supplementary Material

Supplemental Material

## Declarations

**Ethics approval and consent to participate**

Clinical data were recorded and approved by the local ethics committee from both the Second Affiliated Hospital, Zhejiang University School of Medicine (2018-01-F) and the Lishui People’s Hospital (2017-005-01).
