# Supplementary material for: Convergence of carbapenem resistance and hypervirulence in a highly-transmissible ST11 clone of K. pneumoniae: An epidemiological, genomic and functional study
Source: Virulence. 2021 Jan 18;12(1):377–88. doi: 10.1080/21505594.2020.1867468 (PMC7834077; doi:10.1080/21505594.2020.1867468)
Supplement: Supplemental Material [file KVIR_A_1867468_SM9500.docx]

**Supplementary Materials for**

Convergence of carbapenem resistance and hypervirulence in a highly-transmissible ST11 clone of *K. pneumoniae*: an epidemiological, genomic and functional study

Ping Li1#, Qiqiang Liang1#, Wugao Liu2#, Beiwen Zheng3#, Lizhang Liu1#, Wei Wang2, Zhijiang Xu4, Man Huang1*, Youjun Feng1,5, 6*

1 Department of Pathogen Biology & Microbiology and Department of General Intensive Care Unit of the Second Affiliated Hospital, Zhejiang University School of Medicine, Hangzhou, Zhejiang 310058, China

2 Clinical Laboratory of Lishui People’s Hospital, Lishui, Zhejiang 323000, China

3 The First Affiliated Hospital, Zhejiang University School of Medicine, Hangzhou, Zhejiang 310058, China

4 Clinical Laboratory, the Second Affiliated Hospital, Zhejiang University School of Medicine, Hangzhou, Zhejiang 310058, China

5 Non-coding RNA and Drug Discovery Key Laboratory of Sichuan Province, Chengdu Medical College, Chengdu, Sichuan 610500, China

6 College of Animal Sciences, Zhejiang University, Hangzhou, Zhejiang 310058, China

This **SOM** includes Supplementary text; **7** Supplementary tables, and **8** Supplementary figures.

**Supplementary text:**

Detailed information about 18 inpatients admitted into the general ICU of the Second Affiliated Hospital of Zhejiang University

Patient 1:

The patient was admitted to the hospital due to unconsciousness, and CT indicated "bilateral subdural hematoma of the forehead". He was diagnosed to have bilateral frontal subdural hematoma with subarachnoid hemorrhage. Hematoma removal under general anesthesia and bone flap decompression were performed urgently. After the operation, the patient was admitted to ICU for mechanical ventilation, antibiotics, dehydration, etc. High fever occurred repeatedly after he was transferred to the general ward, and then he was transferred to the ICU again due to low oxygen saturation. Subsequent blood culture, urine culture, and sputum culture all indicated CRKP infection. The antibiotic regimen was adjusted to meropenem and tigecycline, followed with polymyxin B. After treatment, the patient gradually recovered and was discharged.

Patient 3:

The patient repeatedly coughed sputum more than 9 years and with aggravated shortness of breath for more than 1 month. He was diagnosed as having acute exacerbation of a chronic obstructive pulmonary disease; II type of respiratory failure. After admission, sulperazone combined with caspofungin and other treatment was given. Subsequently, his sputum culture suggested positive CRKP infection, then, the antibiotic was changed to meropenem. However, the infection was still not under control and blood culture indicated positive CRKP. Secondary hematocytopenia occurred and then the patient's blood pressure was progressively decreased with olivine. He died eventually because of sepsis shock, acute kidney injury, and multiple organ dysfunction.

Patient 4:

The patient had an accident and was sent to the hospital immediately 11 days ago. Emergency CT indicated splenic contusion and laceration accompanied by abdominal effusion and multiple fractures of the left ribs. Emergency treatment was performed with exploratory laparotomy, splenectomy, and gastrostomy. He was indwelled with three drainage tubes in the abdominal cavity and were transferred to ICU. Subsequently, the patient developed the abdominal infection, combined with acute kidney injury and metabolic acidosis. The treatment effect was so unsatisfactory that he was transferred to our ICU. Subsequently, sputum culture of patients suggested CRKP and polymyxin B was added for anti-infection treatment with tigecycline and imipenem. After treatment, the infection was controlled and the patient was discharged after improvement.

Patient 5:

The patient is unconscious as a result of a falling accident. His admitting diagnosis is multiple injuries: brain stem bleeding and subarachnoid hemorrhage. He underwent percutaneous tracheotomy after repeated tube extubation failure due to spinal cord injury. Subsequently, the bilateral ventricular effusion of the patient was increased, and lumbar cistern catheterization and drainage were given. During hospitalization, the patient suffered from ventilator-associated pneumonia, bacteremia and septic shock caused by carbapenems resistance *Acinetobacter baumannii* (CRAB). Sulperazone, meropenem and other antibiotics were successively given. he was treated with plasma infusion to improve the coagulation function and continuous renal replacement therapy (CRRT) to acute kidney injury. However, CT reexamination suggested that the abdominal cavity exudate and effusion, associated with peritonitis. Bedside diagnostic puncture indicated purulent fluid. We recommend surgery rapidly, but family members refused after discussion because of poor prognosis and required automatic discharge. During this period, fecal culture suggested CRKP but colonization was considered combined with clinical manifestation.

Patient 6:

The patient lost consciousness after a traffic accident 26 days ago. He was sent to the local hospital and received craniectomies to remove the osseous flap and hematoma because of bilaterally fixed dilated pupils. After a surgical operation, his pupil back to normal. He was treated in the ICU with tracheotomy antibiotics and other symptomatic and supportive treatment. He was gradually transferred to the recovery department of our hospital. During the treatment, sputum culture showed CRKP which was considered as colonization.

Patient 7:

The patient was admitted to hospital due to quadriplegia caused by trauma and was diagnosed as cervical dislocation as well as cervical spinal cord injury. He coughs yellow sticky sputum and breath with difficulty in the second day after cervical spine fixation. Blood gas analysis indicated type I respiratory failure, so endotracheal intubation was done bedside. The tracheotomy was performed with short-term weaning difficulties. In the meanwhile, intestinal obstruction catheter was implanted with intestinal obstruction. During the treatment, he developed bloodstream infection, liver abscess, and cholecystic infection, which the results of specimen culture indicated CRKP. Sulperazone, meropenem, polymyxin B and tigecycline were successively applied, and catheterization and drainage were performed for liver and gallbladder. Later, diffuse gastrointestinal bleeding occurred, unfortunately. Neither CT examination nor gastroscope and enteroscopy showed obvious bleeding points. Conservative treatments such as drug hemostasis and blood transfusion were only available rather than surgery. He died eventually due to poor treatment of gastrointestinal bleeding, and disseminated intravascular coagulation (DIC), followed by multiple organ dysfunction syndromes (MODS).

Patient 8:

The patient was admitted to hospital due to unconsciousness after sudden dizziness and was diagnosed with spontaneous cerebral hemorrhage. After bilateral ventricular drainage, he was transferred to ICU for symptomatic support. During the hospitalization, he received external ventricular drainage 5 times due to the repeated aggravation of secondary hydrocephalus, but the ventricular drainage tube was repeatedly pulled out due to intracranial infection. He was repeatedly cultured positive with CRAB in intraventricular cerebrospinal fluid. Subsequently, sputum culture and cerebrospinal fluid culture repeatedly suggested CRKP. Although polymyxin B combined with sulperazone were used, the drug-resistant bacteria continued to exist. After the last operation, the patient was transferred to ICU for treatment due to a difficult recovery. The patient's vital signs were stable and transferred to the general ward. But the next day, the patient appeared in a deep coma again and died subsequently because the family members gave up treatment.

Patient 9:

The patient was hospitalized due to sudden unconsciousness and was diagnosed as spontaneous cerebral hemorrhage. He was unable to undergo emergency surgery due to unstable circulation. After intensive treatment, the patient's vital signs gradually stabilized, but he was still in a coma. Then he experienced a sudden decrease in oxygen saturation during subsequent treatment, and pulmonary arteriography suggested a pulmonary embolism. Anticoagulant treatment is needed, but the patient is still in the peak period after cerebral hemorrhage which is a contraindication for anticoagulant treatment. So we had to give up anticoagulant therapy. After treatment, he gradually stabilized and was transferred to neurosurgery for further treatment. After 4 days, he was hyperthermia again, cough sputum and dyspnea. After endotracheal intubation, the patient was transferred to ICU again. After admission, sputum culture suggested that CRKP and meropenem were given to treat pneumonia. Subsequently, the symptoms of pneumonia were controlled. Because of long-term coma, the family gave up treatment and asked for automatic discharge. The prognosis was not good, but it was not caused by infection.

Patient 10:

The patient was hospitalized due to the weakness of the right limb and vomiting. He was diagnosed as cerebral hemorrhage of the left basal ganglia and received craniotomy hematoma removal in emergency surgery. Although tracheotomy was done because of difficult weaning of ventilator, he recovered well in ICU and transferred to the general ward, followed by rehabilitation treatment. During the period, CRKP was positive in sputum culture but was regarded as colonization.

Patient 11:

The patient was hospitalized in the rheumatology department for systemic lupus erythematosus (SLE). She was in a state of hypercoagulation with extended glucocorticoid therapy. During the hospitalization, a shock occurred suddenly. Emergency CT pulmonary arteriography suggested pulmonary hypertension and pulmonary embolism was considered. After she was transferred to ICU for intensive care, her vital signs were stabilized gradually. CRKP was positive in sputum culture but was regarded as colonization.

Patient 12:

The patient had sudden unconsciousness and was diagnosed as spontaneous cerebral hemorrhage in the other hospital. Craniotomy hematoma removal was performed in emergency surgery. The patient developed secondary epilepsy, followed by recurrent basal ganglia hemorrhage and pneumonia after he removed to the general ward. In order to further treatment, he was transferred to our ICU. During the treatment, sputum culture was positive for CRKP, but we considered it as colonizing bacteria

Patient 13:

The patient was diagnosed as moyamoya disease and spontaneous ventricular hemorrhage due to sudden severe headache accompanied by unconsciousness. She was transferred to ICU after emergency treatment of external ventricular drainage. Intracranial infection and ventilator-associated pneumonia distressed for the patient after admission to the ICU. She was improved with effective antibiotics and symptomatic support therapy. CRKP was positive in her urine culture but was considered as colonization.

Patient 14:

The patient was admitted to the hospital due to sudden chest tightness and was diagnosed as Stanford type A arterial dissection. He was transferred to ICU after ascending aorta and aortic replacement under emergency surgery. He suffered from acute kidney injury, thrombotic thrombocytopenic purpura and cardiac embolism caused by pericardial effusion in the hospital and improved through rescue and close monitoring. Due to pneumonia caused by CRKP and respiratory failure, the patient had difficulty in weaning. Subsequently, the patient developed severe bloodstream infection and mediastinal infection, but the culture indicated CRAB. Imipenem and tigecycline were given successively to improve infection symptoms. After active treatment, the patient's condition gradually stabilized and discharged eventually.

Patient 16:

The patient was hospitalized due to pain and discomfort in the neck, chest, and back. Pathological biopsy at the thoracic tumor site indicated metastatic low-differentiated carcinoma, which may come from urinary system possibly. Thoracic tumor resections were performed two times within 10 days. During the hospitalization, the patient's urine culture indicated CRKP, but he had no obvious symptoms.

Patient 17:

The patient was admitted to hospital due to unconsciousness and was diagnosed as spontaneous cerebral infarction. He was transferred to our hospital after emergency thrombolytic therapy. Anticoagulant drugs must be discontinued because of subsequently suffered cerebral hemorrhage after cerebral infarction. Subsequently, a large area of cerebral infarction again leads to mechanical ventilation and other symptomatic treatment. Despite ventilator-associated pneumonia with CRAB, his condition was stable and successful extubation. Then he was transferred to the general ward and tigecycline and biapenem were used for pneumonia with CRAB and CRKP which we think it as colonization. He was discharged after improvement.

Patient 18:

During the CT examination, the patient suffered sudden respiratory and cardiac arrest. After 5 minutes of cardiopulmonary resuscitation, the patient was transferred to ICU for intensive monitoring. After admission to ICU, the patient developed stress ulcer and coagulation dysfunction, which gradually stabilized after treatment. A week later, the patient presented with high fever and sputum culture as CRKP. Meropenem was used for antibiotic treatment. Tracheal intubation was removed when he was gradually sober. Reexamination of head MRI after endotracheal intubation revealed encephalopyosis, which may be the cause of cardiac arrest. Right temporal lobe lesion biopsy was performed surgically, and encephalopyosis was considered in combination with pathological results. The patient recovered stably after surgery and was discharged after improvement

Patient 19:

The patient was in a coma at the local hospital and underwent head CT examination, which indicated cerebral hemorrhage in the right frontal-parietal lobe. Sudden cardiac arrest occurred after hospitalization and spontaneous cardiac recovery occurred 18 minutes after cardiopulmonary resuscitation. He was transferred to the ICU of our hospital and diagnosed as cerebral hemorrhage, secondary epilepsy of ischemic and hypoxic encephalopathy after cardiopulmonary resuscitation. Mechanical ventilation and mild hypothermia therapy were given after admission. Ischemia and hypoxic encephalopathy led to difficulty in weaning and tracheotomy as planned. However, we had to give up tracheotomy due to the patient's obesity and difficulty in tracheal exposure. The patient had long-term endotracheal intubation, obvious falling volume pneumonia, and aggravated pulmonary infection. CRKP was found in blood culture and sputum culture successively with meropenem and tigecycline treatments. His mental recovery was poor and the family members required automatic discharge.

**Supplementary tables**

**Table S1** Clinical isolates of *K. pneumoniae* reported in this study

| Stock no. | Patients | Description | Sources | Date |  |
| --- | --- | --- | --- | --- | --- |
| The Second Affiliated Hospital, Zhejiang University, Hangzhou City, China | | | | | |
| FYJ1165 | Patient 1 | K185/pK185_*rmpA2* & pK185_KPC | Sputum | 09/2017 |  |
| FYJ1167 | Patient 3 | K187/pK187_*rmpA2* & pK187_KPC | Blood | 08/2017 |  |
| FYJ1171 | Patient 4 | K192/pK192_*rmpA2* & pK192_KPC | Sputum | 10/2017 |  |
| FYJ1174 | Patient 5 | K195/pK195_*rmpA2* & pK195_KPC | Feces | 08/2017 |  |
| FYJ1175 | Patient 6 | K196/pK196_KPC | Sputum | 10/2017 |  |
| FYJ1187 | Patient 7 | K198/pK198_*rmpA2* | Feces | 11/2017 |  |
| FYJ1188 | Patient 7 | K199/pK199_*rmpA2* & pK199_KPC | Ascites | 11/2017 |  |
| FYJ1209 | Patient 8 | K230/pK230_*rmpA2* & pK230_KPC | Perineum | 08/2017 |  |
| FYJ1211 | Patient 8 | K232/pK232_*rmpA2* & pK232_KPC | Oral | 08/2017 |  |
| FYJ1213 | Patient 8 | K234/pK234_*rmpA2* & pK234_KPC | CSF | 08/2017 |  |
| FYJ1214 | Patient 17 | K235/pK235_*rmpA2* & pK235_KPC | Sputum | 07/2017 |  |
| FYJ1216 | Patient 18 | K237/pK237_KPC | Sputum | 11/2017 |  |
| FYJ1218 | Patient 19 | K239/pK239_*rmpA2* & pK239_KPC | Blood | 11/2017 |  |
| Lishui People’s Hospital, Lishui City, China | | | | | |
| FYJ1177 | Patient 20 | K202/pK202_*rmpA2* | Urine | 1/2017 |  |
| FYJ1179 | Patient 21 | K204/pK204_*rmpA2* & pK204_KPC | Urine | 6/2017 |  |
| FYJ1190 | Patient 22 | K211/pK211_*rmpA2* & pK211_KPC | Blood | 10/2017 |  |
| FYJ1191 | Patient 23 | K212/pK212_KPC | Urine | 3/2017 |  |
| FYJ1192 | Patient 24 | K213/pK213_*rmpA2* & pK213_KPC | Urine | 9/2017 |  |
| FYJ1194 | Patient 25 | K214/pK214_*rmpA2* & pK214_KPC | Urine | 2/2017 |  |

**Table S5** Primers used in this study

| Primers | Primer sequences |
| --- | --- |
| Virulence genes | |
| *rmpA*-F | 5’- ATG GCC TAA AGC AGT TAA CTG-3’ |
| *rmpA*- R | 5’- CTA AAT ACT TGG CAT GAG CCA-3’ |
| *rmpA2*-F | 5’- ATT TAC TTT ATG TGC AAT AAG G-3’ |
| *rmpA2*–R | 5’- CTA GGT ATT TGA TGT GCA CCA-3’ |
| *IutA*–F | 5’- AAA GTA TAC GCT TTG GGC TCT-3’ |
| *IutA*–R | 5’- AGA GTA GTT CAG ACC AAA GGT-3’ |
| *IucA*-F | 5’- AGC AGG AAT GGT GCC AGG AGC-3’ |
| *IucA*-R | 5’- CCG AGG GAT CGA CGA TGG TGT-3’ |
| *IroN*-F | 5’- ATG AGA ATT AAC AAG TCC TCT-3’ |
| *IroN*-R | 5’- TAT AGG TAC AGC ACC TTC TG-3’ |
| Antibiotic resistance genes | |
| *bla*_KPC_-F | 5’- CAG CTC ATT CAA GGG CTT TCT T-3’ |
| *bla*_KPC_-R | 5’- GCA GAC TCC AGC CTA AAT GTG-3’ |
| *bla*_NDM_-F | 5’- GCT ACA GTG AAC CAA ATT AAG -3’ |
| *bla*_NDM_-R | 5’- AAT GGC TCA TCA CGA TCA TG-3’ |
| *bla*_VIM_-F | 5’- GTA GTT TAT TGG TCT ACA TGA C-3 |
| *bla*_VIM_-R | 5’- TTG TGT GCT TTG ACA ACG TT-3’ |
| *bla*_IMP_-F | 5’- GTA GTT TAT TGG TCT ACA TGA C-3’ |
| *bla*_IMP_-R | 5’- AGT TTC AAG AGT GAT GCG TCT-3’ |
| *bla*_OXA48_-F | 5’- AGT TTC AAG AGT GAT GCG TCT-3’ |
| *bla*_OXA48_-R | 5’- ATG CGT GTA TAG CCT TAT CG-3’ |
| *mcr-1*-F | 5’-ATG ATG CAG CAT ACT TCT GTG-3’ |
| *mcr-1*-R | 5’-TCA GCG GAT GAA TGC GGT G-3’ |
| MLST | |
| *rpoB*-F | 5’-GGC GAA ATG GCW GAG AAC CA-3’ |
| *rpoB*-R | 5’-GAG TCT TCG AAG TTG TAA CC-3’ |
| *gapA*-F | 5’-TGA AAT ATG ACT CCA CTC ACG G-3’ |
| *gapA*-R | 5’-CTT CAG AAG CGG CTT TGA TGG CTT-3’ |
| *mdh*-F | 5’-CCC AAC TCG CTT CAG GTT CAG-3’ |
| *mdh*-R | 5’-CCG TTT TTC CCC AGC AGC AG-3’ |
| *pgi*-F | 5’-AGA GAA AAA CCT GCC TGT ACT GCT-3’ GGC-3’ |
| *pgi*-R | 5’-CGC GCC ACG CTT TAT AGC GGT TAA T-3’ |
| *phoE*-F | 5’-ACC TAC CGC AAC ACC GAC TTC TTC GG-3’ |
| *phoE*-R | 5’-TGA TCA GAA CTG GTA GGT GAT-3’ |
| *infB*-F | 5’-CTC GCT GCT GGA CTA TAT TCG-3’ |
| *infB*-R | 5’-CGC TTT CAG CTC AAG AAC TT-3’ |
| *tonB*-F | 5’-CTT TAT ACC TCG GTA CAT CAG GTT-3’ |
| *tonB*-R | 5’-ATT CGC CGG CTG RGC RGA GAG-3’ |

**Table S6** Overview of plasmids and its *K. pneumoniae* hosts

| No. | *K. pneumoniae* | ST | Plasmid size (kb) | |
| --- | --- | --- | --- | --- |
|  |  |  | KPC-2 | *rmpA2* |
| 1 | K187 | ST11 | ~120 | ~180 |
| 2 | K192 | ST11 | NA | ~180 |
| 3 | K195 | ST11 | ~120 | ~180 |
| 4 | K198 | ST11 | NA | ~180 |
| 5 | K199 | ST11 | ~120 | ~180 |
| 6 | K202 | ST11 | NA | ~180 |
| 7 | K204 | ST11 | ~120 | ~180 |
| 8 | K230 | ST11 | ~120 | ~180 |
| 9 | K232 | ST11 | ~120 | ~180 |
| 10 | K234 | ST11 | ~180 | ~180 |
| 11 | K235 | ST11 | ~120 | ~180 |
| 12 | K239 | ST11 | ~120 | ~180 |
| 13 | K212 | ST11 | ~60 | / |
| 14 | K213 | ST11 | ~60 | / |
| 15 | K237 | ST11 | ~60 | / |
| 16 | K185 | ST11 | NA | ~180 |
| 17 | K214 | ST11 | ~180 | ~180 |
| 18 | K211 | ST11 | ~80 | ~180 |
| 19 | K196 | ST449 | ~90 | / |

**Table S7** Profile of antimicrobial resistance determinants carried in different KPC-2-harboring plasmids

| Plasmids | Antibiotic resistance genes |
| --- | --- |
| pK185_KPC | KPC-2, TEM-1B, CTX-M-65, SHV-12, *rmtB*, *catA2* |
| pK187_KPC | KPC-2, TEM-1B, CTX-M-65, *rmtB*, *catA2* |
| pK192_KPC | KPC-2, TEM-1B, *rmtB*, *catA2* |
| pK195_KPC | KPC-2, TEM-1B, CTX-M-65, *rmtB* |
| pK196_KPC | KPC-2, *aac(3)-IId*, *qnrS1*, *sul1*, *tet(A)*, *dfrA1* |
| pK198_KPC | KPC-2, TEM-1B, CTX-M-65, *rmtB* |
| pK199_KPC | KPC-2, TEM-1B, CTX-M-90, *rmtB* |
| pK202_KPC | KPC-2, TEM-1B, CTX-M-65, *rmtB*, *catA2* |
| pK204_KPC | KPC-2, TEM-1B, CTX-M-65, *aph(3')-Ia*, *rmtB*, *catA2* |
| pK211_KPC | KPC-2 |
| pK212_KPC | KPC-2 |
| pK213_KPC | KPC-2 |
| pK214_KPC | KPC-2 |
| pK230_KPC | KPC-2, TEM-1B, CTX-M-65, *rmtB* |
| pK232_KPC | KPC-2, CTX-M-65 |
| pK234_KPC | KPC-2 |
| pK235_KPC | KPC-2, TEM-1B, CTX-M-65, *rmtB*, *catA2* |
| pK237_KPC | KPC-2 |
| pK239_KPC | KPC-2, TEM-1B, CTX-M-65, *rmtB* |

The antibiotic resistance genes include KPC-2 carbapenemase, four types of β-lactamases (TEM-1B, CTX-M-65, CTX-M-90 and SHV-12), aminoglycoside resistance gene *rmtB* and phenicol resistance gene *catA2*.

**Supplementary figures**


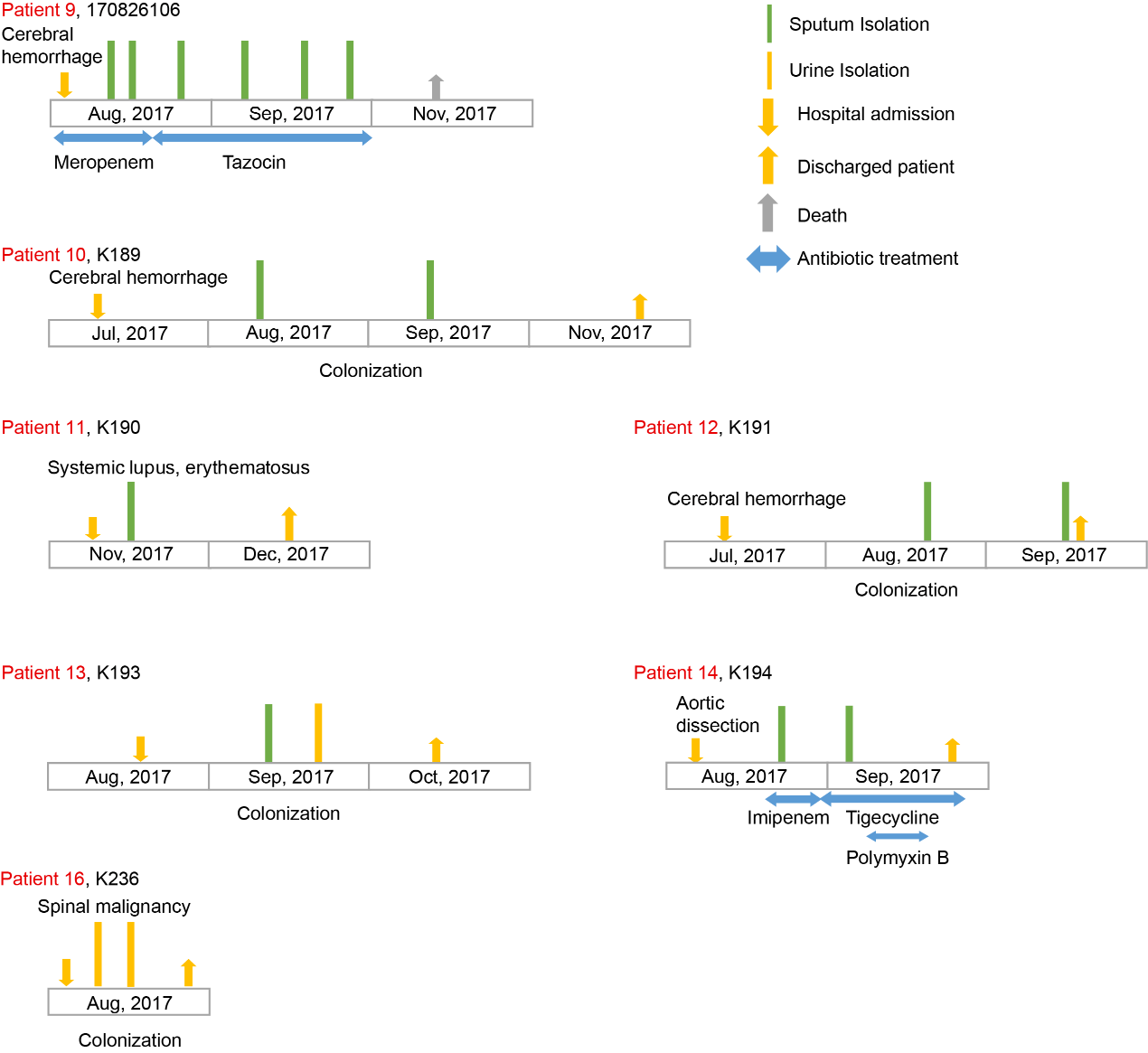


**Fig. S1** Clinical characterization of seven patients (from Patient 9 to Patient 14, and Patient 16) admitted to the Second Affiliated Hospital, Zhejiang University School of Medicine in 2017


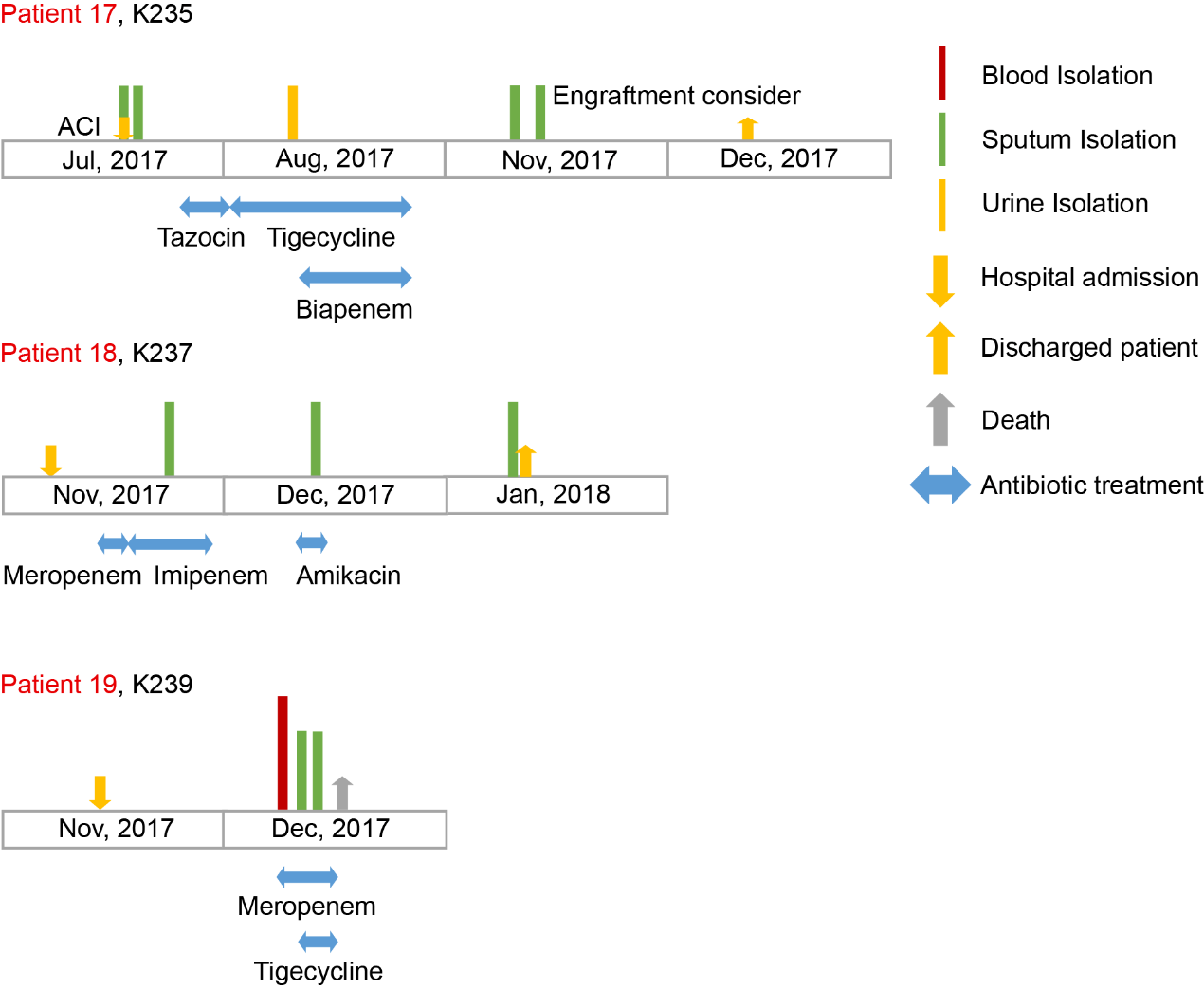


**Fig. S2** Clinical description of the three remaining patients (Patient 17, Patient 18 & Patient 19) admitted to the Second Affiliated Hospital, Zhejiang University School of Medicine in 2017


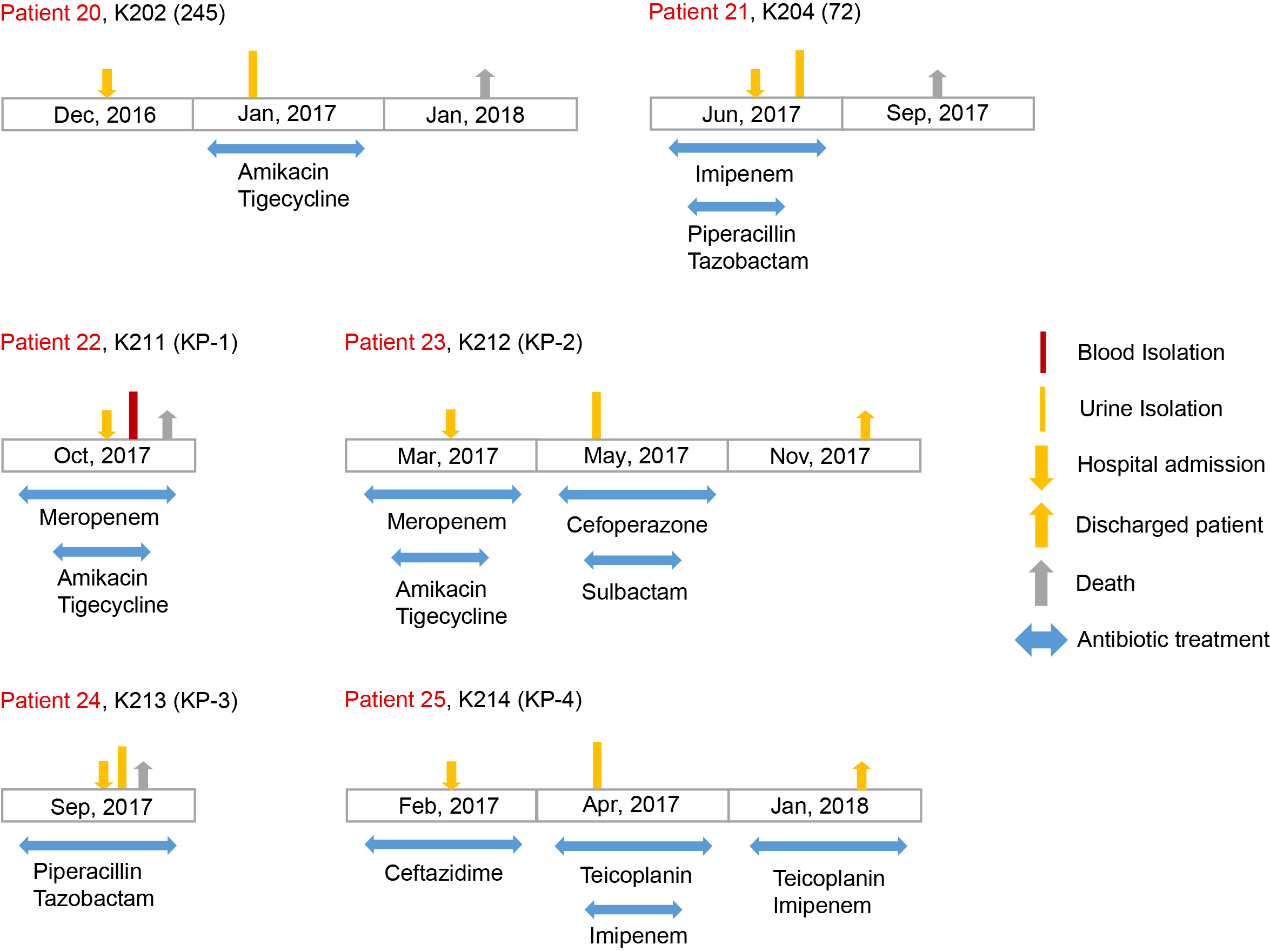


**Fig. S3** Clinical characterization of six patients (Patient 20, to Patient 25) admitted to the Lishui People’s Hospital, Lishui City, Zhejiang, in 2017


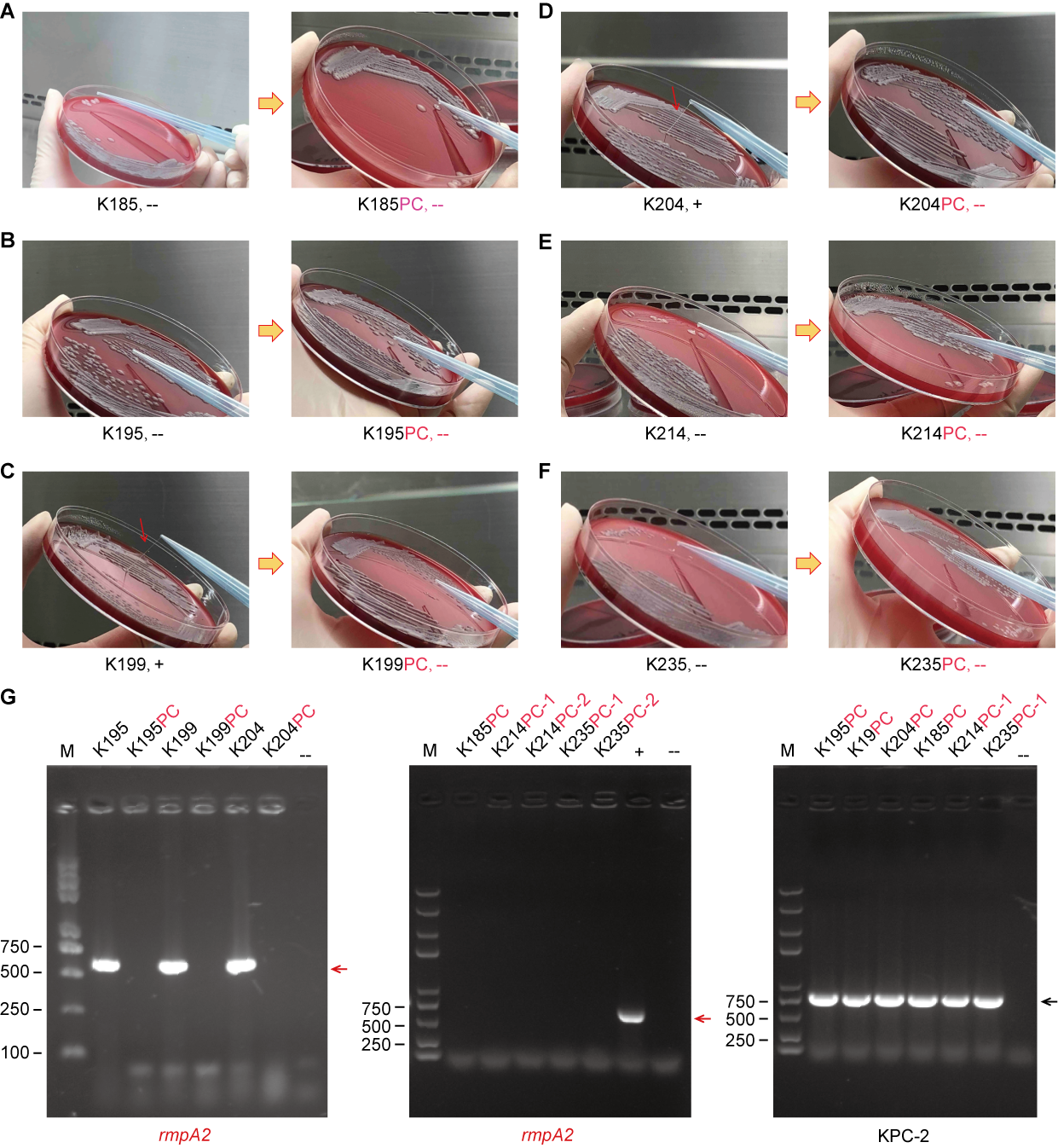


**Fig. S4** Microbial and molecular evidence for the *rmpA2*-plus virulence plasmid curing

**A-B.** String tests for the two *rmpA2*-positive isolates of *K. pneumoniae* (K185 and K195) without detectable hypermucoviscosity

**C-D.** String tests suggested that the *rmpA2*-positive virulence plasmid curing results in the loss of hypermucoviscosity in the two clinical isolates of *K. pneumoniae* (K199 and K204)

**E-F.** Regardless of the *rmpA2*-positive virulence plasmid curing, the two clinical strains (K214 and K235) of *K. pneumoniae* are negative in string tests

**G.** Use of *rmpA2*-, and KPC-2-specific PCR assays to validate the clincial strains with the loss of *rmpA2*-virulence plasmid, but retaining the KPC-2-bearing plasmid.

Designations: M, DNA marker; PC, plasmid curing. The symbols plus (+) and minus (--) separately represnt the presence (or loss) of phenotypic hypermucoviscosity in the string tests.


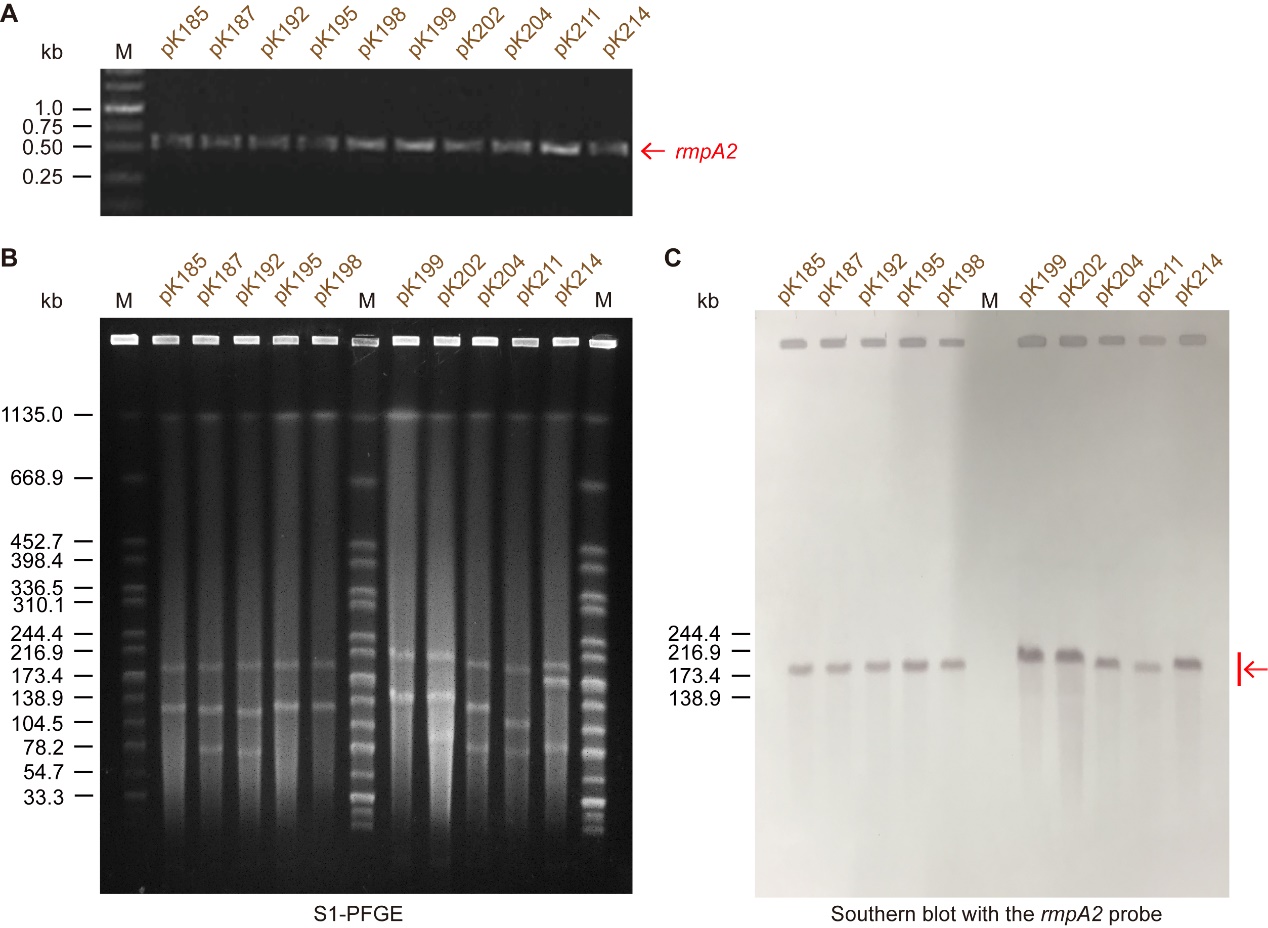


**Fig. S5** Genetic analyses of the *rmpA2*-containing plasmids from clinical isolates of virulent *K. pneumoniae*

**A.** PCR assays for the presence of *rmpA2* in the plasmids from clinical isolates of virulent *K. pneumoniae*

**B.** S1-PFGE analyses of the *rmpA2*-harboring plasmids

**C.** Use of Southern blot to determine the physical size of the *rmpA2*-bearing plasmids

Designations: M refers to DNA marker (DL5000 in **Panel A** and the digestion products of *Salmonella enterica* with XhoI in **Panels B**&**C**). Letters (in brown) denote those plasmids from the isolates of Second Affiliated Hospital, Zhejiang University, Hangzhou, China.


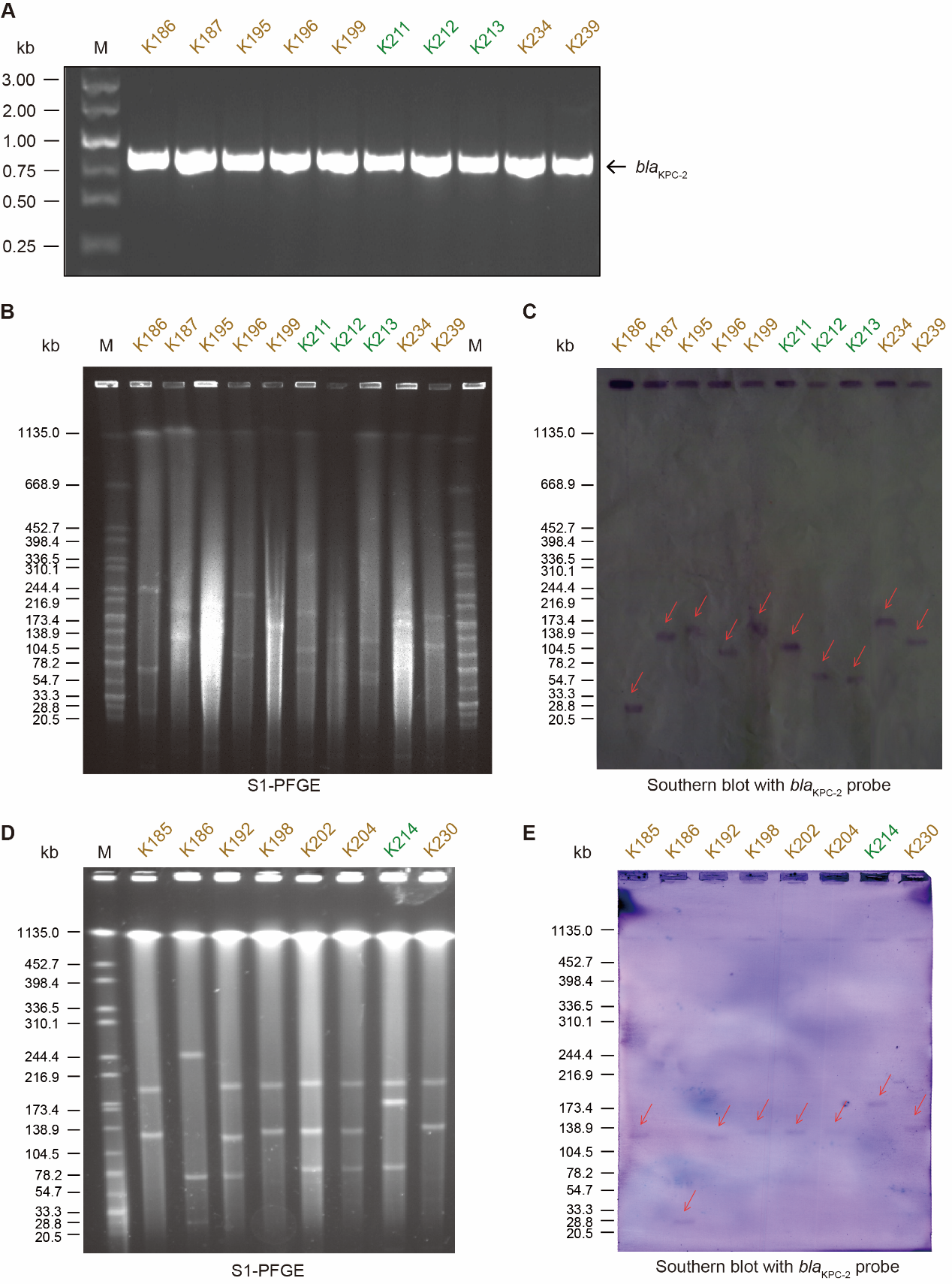


**Fig. S6** Genetic analyses of the KPC-2 carbapenemase-producing plasmids from clinical isolates of virulent *K. pneumoniae*

**A.** PCR assays of clinical isolates of virulent *K. pneumoniae* with a pair of *bla*_KPC-2_-specific primers

**B&D.** S1-PFGE profile of the *bla*_KPC-2_-carrying plasmids

**C&E.** Use of Southern blot to probe the diversified size of the *bla*_KPC-2_-harboring plasmids

Designations: M refers to DNA marker (DL5000 in **Panel A** and the digestion products of *Salmonella enterica* genomic DNA with XhoI in **Panels B**-**E**).

Letters (in brown) denote those plasmids from the isolates of Second Affiliated Hospital, Zhejiang University, whereas the green letters refer to plasmids from the isolates of Lishui’s People Hospital, Zhejiang, China.


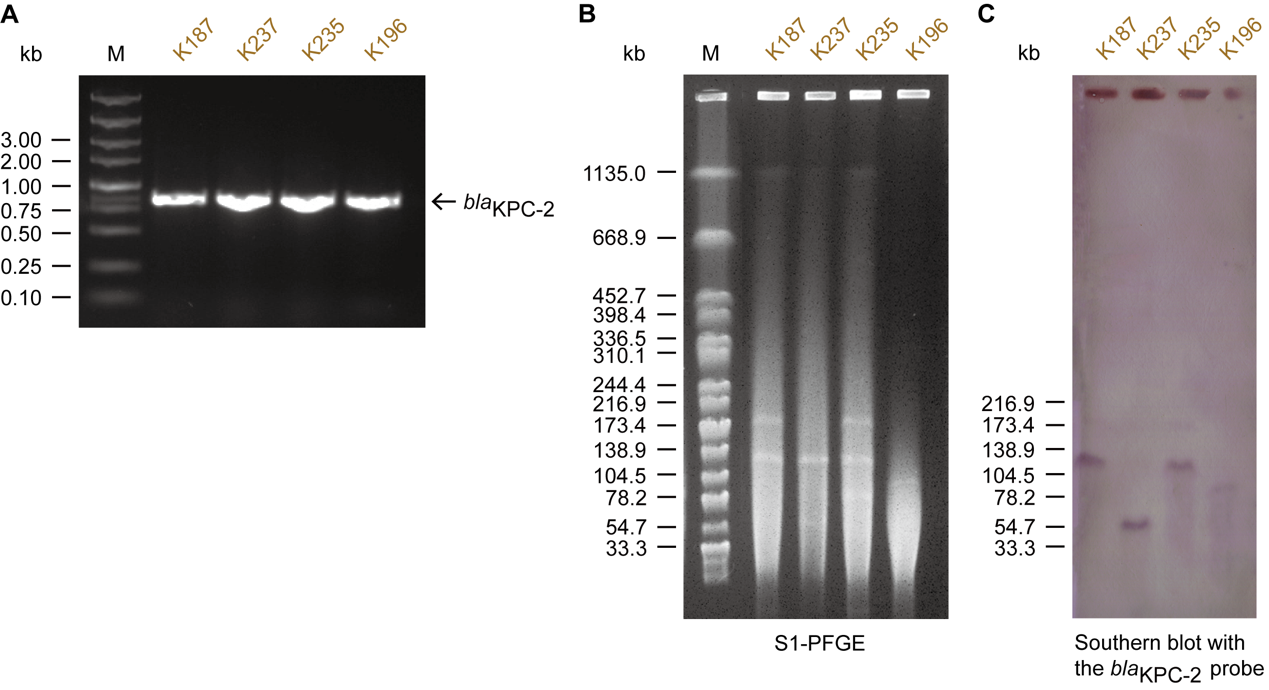


**Fig. S7** Molecular analyses of the remaining 4 *bla*_KPC-2_-containing plasmids from clinical isolates of virulent *K. pneumoniae*

**A.** The PCR detection for the presence of *bla*_KPC-2_ in four clinical isolates of virulent *K. pneumoniae*

**B.** S1-PFGE profile of the four KPC-2-producing plasmids

**C.** Southern blotting analyses of the *bla*_KPC-2_-harboring plasmids

Designations: M refers to DNA marker (DL5000 in **Panel A** and the digestion products of *Salmonella enterica* with XhoI in **Panels B**&**C**).

Letters (in brown) denote those plasmids from bacterial isolates of the Second Affiliated Hospital, Zhejiang University, Hangzhou, China.


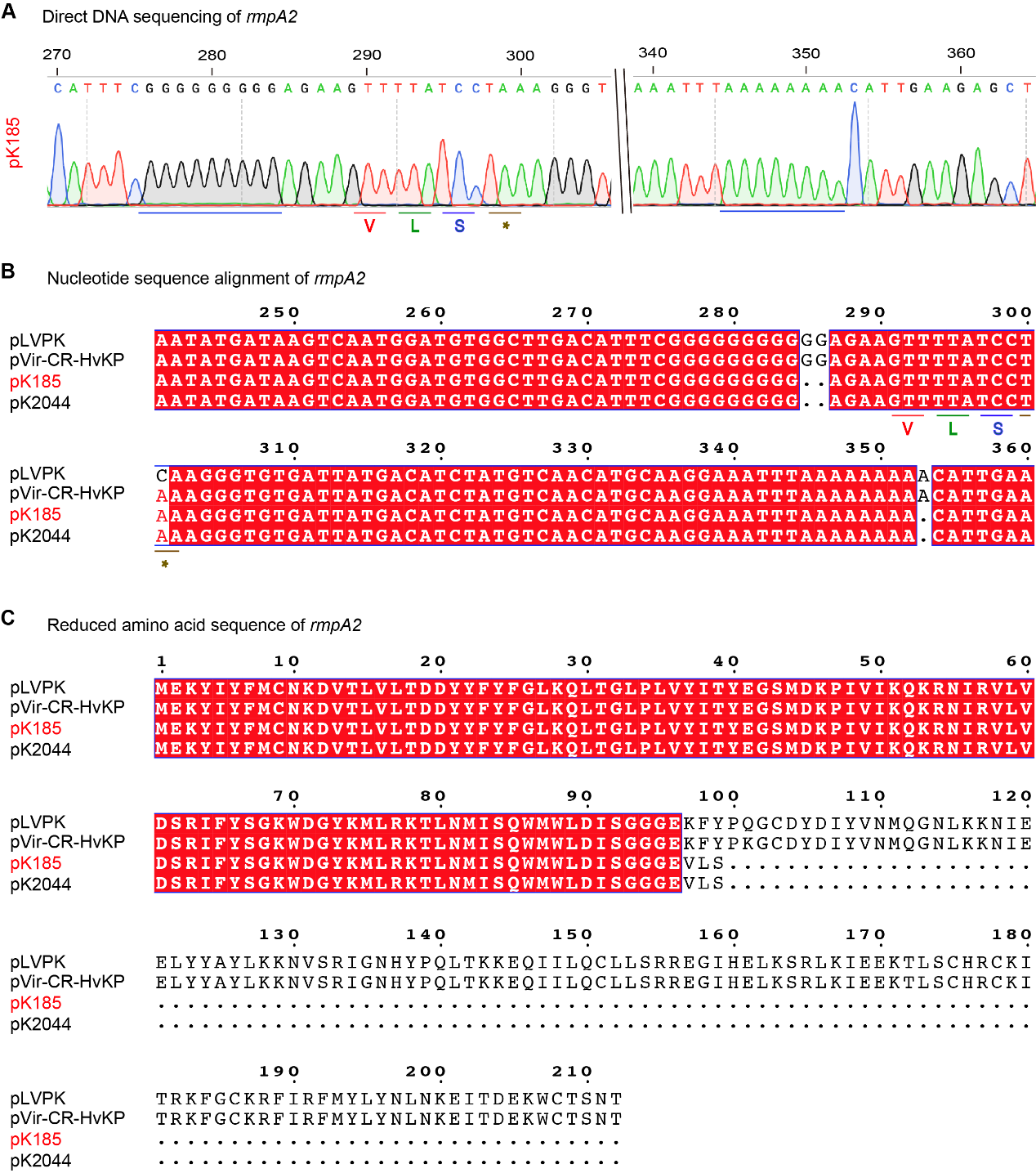


**Fig. S8** Sequencing and analysis of the virulence regulator-encoding gene *rmpA2*

**A.** Sanger sequencing of *rmpA2*

A representative result of direct DNA sequencing of *rmpA2* PCR products is given here.

**B.** Nucleotide acid sequence alignment of *rmpA2*

Two positions of nucleotide deletions were detected in our samples, namely the two residues of G285-G286, and A353. This results in an truncated and premature version of RmpA2 in the length of 99aa

**C.** Amino acid sequence alignment of RmpA2
